# Targeting claudin‐overexpressing thyroid and lung cancer by modified *Clostridium perfringens* enterotoxin

**DOI:** 10.1002/1878-0261.12615

**Published:** 2020-01-08

**Authors:** Anna Piontek, Miriam Eichner, Denise Zwanziger, Laura‐Sophie Beier, Jonas Protze, Wolfgang Walther, Sarah Theurer, Kurt Werner Schmid, Dagmar Führer‐Sakel, Jörg Piontek, Gerd Krause

**Affiliations:** ^1^ Leibniz‐Forschungsinstitut für Molekulare Pharmakologie (FMP) Berlin Germany; ^2^ Institute of Clinical Physiology / Nutritional Medicine, Medical Department Division of Gastroenterology, Infectiology, Rheumatology, Charitè – Universitätsmedizin Berlin Germany; ^3^ Department of Endocrinology, Diabetes and Metabolism and Clinical Chemistry – Division of Laboratory Research University Hospital Essen Germany; ^4^ Experimental and Clinical Research Center Charitè and Max‐Delbrück‐Center for Molecular Medicine Berlin Germany; ^5^ Institute of Pathology University Hospital Essen Germany

**Keywords:** claudins, *Clostridium perfringens* enterotoxin, directed mutagenesis, lung cancer, necrosis, thyroid cancer

## Abstract

*Clostridium perfringens* enterotoxin (CPE) can be used to eliminate carcinoma cells that overexpress on their cell surface CPE receptors – a subset of claudins (e.g., Cldn3 and Cldn4). However, CPE cannot target tumors expressing solely CPE‐insensitive claudins (such as Cldn1 and Cldn5). To overcome this limitation, structure‐guided modifications were used to generate CPE variants that can strongly bind to Cldn1, Cldn2 and/or Cldn5, while maintaining the ability to bind Cldn3 and Cldn4. This enabled (a) targeting of the most frequent endocrine malignancy, namely, Cldn1‐overexpressing thyroid cancer, and (b) improved targeting of the most common cancer type worldwide, non‐small‐cell lung cancer (NSCLC), which is characterized by high expression of several claudins, including Cldn1 and Cldn5. Different CPE variants, including the novel mutant CPE‐Mut3 (S231R/S313H), were applied on thyroid cancer (K1 cells) and NSCLC (PC‐9 cells) models. *In vitro*, CPE‐Mut3, but not CPEwt, showed Cldn1‐dependent binding and cytotoxicity toward K1 cells. For PC‐9 cells, CPE‐Mut3 improved claudin‐dependent cytotoxic targeting, when compared to CPEwt*. In vivo,* intratumoral injection of CPE‐Mut3 in xenograft models bearing K1 or PC‐9 tumors induced necrosis and reduced the growth of both tumor types. Thus, directed modification of CPE enables eradication of tumor entities that cannot be targeted by CPEwt, for instance, Cldn1‐overexpressing thyroid cancer by using the novel CPE‐Mut3.

## Graphical abstract

*Clostridium perfringens* enterotoxin (CPE) is used to target carcinomas overexpressing a claudin subset serving as CPE receptors. CPE‐based pores in membrane cause cell death. Structure‐guided CPE modifications (CPE‐S231R/S313H) enabled also claudin‐1 binding and growth reduction of claudin‐1‐expressing papillary thyroid carcinoma (mouse xenotransplants) that could not be targeted by CPEwt. Furthermore, CPE‐S231R/S313H improved targeting of lung cancer (NSCLC) expressing multiple claudins.

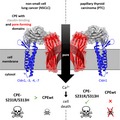

**Abbreviations**
cCPE: C‐terminal domain of CPE
CDX: cell line‐derived xenotransplant
Cldn: claudins
CPE: *Clostridium perfringens* enterotoxin
HE: hematoxylin and eosin
Mut: mutant
NSCLC: non‐small‐cell lung cancer
PTC: papillary thyroid carcinoma
TJs: tight junctions
TV: tumor volume
wt: wild‐type

## 1 Introduction

Thyroid cancer is the most common endocrine malignancy. Papillary thyroid carcinoma (PTC), a differentiated type of thyroid cancer, is the most common histological subtype accounting for approximately 70% of thyroid cancer cases (Wartofsky, 2010). Often, surgery is the most successful treatment for PTC patients and is normally associated with a good prognosis. However, approximately 20–25% of patients develop distant metastases (most in lung and bone) of PTC and have a worse prognosis, since advanced PTC often does not respond to conventional radioactive iodine therapy (radioactive iodine refractory thyroid cancer).

Lung cancer (both small‐cell and non‐small‐cell lung cancer – NSCLC) is the most common cancer (80–85% of all cases) and is the most common cause of death from cancer worldwide. In addition, approximately 50–70% of patients with lung adenocarcinoma, one type of NSCLC, after surgery relapse within one year and their cancer cells acquire a chemoresistant phenotype (Ramalingam and Belani, 2008). Targeted drugs, such as angiogenesis inhibitors, epidermal growth factor receptor inhibitors, anaplastic lymphoma kinase inhibitors, and immunotherapy drugs, that act via T cells, eventually can shrink tumors for several months only and are associated with side effects (Silva *et al.*, 2017). Therefore, treatment of radioiodine refractory thyroid cancer and chemoresistant NSCLC remains challenging and new targets and therapeutic substances are desperately needed.

One promising strategy for targeted cancer therapy is delivery of foreign toxic molecules specifically to tumor cells (Walther *et al.*, 2012). Bacterial toxins, as attractive candidates, demonstrated efficient cell‐killing capacity in several *in vitro* and *in vivo* studies (Dang *et al.*, 2001; Michl and Gress, 2004; Morin, 2005). Employment of pore‐forming bacterial toxins, such as *Clostridium perfringens* enterotoxin (CPE) (Minton, 2003; Walther *et al.*, 2012), enables specific targeting of their receptors, which are frequently expressed on cancer cells’ surface. Thereby, the strategy has a great potential to target cancer cells, while minimizing side effects on normal cells.

*Clostridium perfringens* enterotoxin, mainly associated with food poisoning, is released by anaerobic Gram‐positive *C. perfringens* type A strains. CPE is a β‐pore‐forming toxin, consisting of 319 amino acids (35 kDa) and two functional domains with a known structure (pdb: http://www.rcsb.org/pdb/search/structidSearch.do?structureId=2XH6, Briggs *et al.*, 2011, pdb: http://www.rcsb.org/pdb/search/structidSearch.do?structureId=3AM2, Kitadokoro *et al.*, 2011). CPE‐mediated cytotoxicity has been studied and described thoroughly. The procedure starts with CPE binding to its receptors (claudins) on the plasma membrane via its C‐terminal domain (cCPE) (McClane, 2001; Veshnyakova *et al.*, 2010), whereas the N‐terminal domain inserts into membrane and participates in cytotoxic pore formation allowing rapid Ca^2+^‐influx leading to cell death (Chakrabarti and McClane, 2005; Eichner *et al.*, 2017). In contrast to full length CPE, the C‐terminal domain of CPE (amino acids 194–319, cCPE) is not toxic (Kokai‐Kun and McClane, 1997), but maintains binding to claudins (McClane, 2001). Claudins (Cldn, 27 family members) constitute tight junctions (TJs) which regulate paracellular permeability across epithelia. CPE interacts only with a subset of claudins. It binds with high affinity (*K*_d_ ~ 10 nm) to Cldn3, Cldn4, Cldn6, Cldn7, and Cldn9; with medium affinity to Cldn8, Cldn14, and Cldn19; and with low affinity to Cldn1 and Cldn2, but does not bind to Cldn5, Cldn10 to Cldn13, Cldn15 to Cldn18, and Cldn20 to Cldn24 (Bocsik *et al.*, 2016; Veshnyakova *et al.*, 2010).

In carcinoma cells, claudins are usually mislocalized out of TJs (extrajunctional) and often deregulated (Kwon, 2013), thereby not necessarily contributing to TJ sealing and paracellular barrier regulation. Many epithelial tumors exhibit increased claudin expression (Kominsky *et al.*, 2004; Long *et al.*, 2001; Lu *et al.*, 2013; Michl *et al.*, 2001; Santin *et al.*, 2007; Soini and Talvensaari‐Mattila, 2006), which has been discussed as potential marker in oncology (Long *et al.*, 2001). Claudin upregulation is assumed to benefit tumor progression by promoting cell migration, invasion, and metastasis (Jian *et al.*, 2015). In addition to such aberrant expression, extrajunctional mislocalization of claudin proteins may contribute to their role in tumorigenesis (Boireau *et al.*, 2007).

In case of thyroid cancer (Zwanziger *et al.*, 2015) and NSCLC, overexpression (Jung *et al.*, 2009; Sun *et al.*, 2016) and extrajunctional mislocalization of Cldn1, with impact on cell–cell contacts and proliferation, is a specific hallmark. Expression of Cldn1 was demonstrated in PTC, papillary microcarcinoma primary tumors, and lymph node metastases (Nemeth *et al.*, 2010) and is assumed to be a prognostic factor for patients with lung adenocarcinoma (Sun *et al.*, 2016). This makes Cldn1 a promising target for therapeutic approaches.

Several studies have suggested CPE as a biologic for treatment of tumors overexpressing claudins (Black *et al.*, 2015; Kominsky *et al.*, 2007; Morin, 2007; Veshnyakova *et al.*, 2010). Dependence of CPE‐mediated cytotoxicity on expression of CPE‐sensitive claudins was shown in numerous studies (Eichner *et al.*, 2017; Fujita *et al.*, 2000; Robertson *et al.*, 2010; Sonoda *et al.*, 1999), including claudin knockdowns (Casagrande *et al.*, 2011; Pahle *et al.*, 2017; Tanaka *et al.*, 2018).

However, employment of wild‐type CPE (CPEwt) for cancer treatment is restricted to carcinomas, which express CPEwt‐binding claudins (e.g., Cldn3, Cldn4). In addition, extrajunctional CPE‐sensitive claudin molecules are also present on normal epithelia, at least to a minor extent. This can result in side effects during CPE treatment. To overcome these difficulties, we modified CPE for the first time to adjust its claudin‐binding properties to the claudin expression profile of certain cancer types containing claudin subtypes that are not bound by CPEwt.

Here, we demonstrate expansion of CPE‐based oncoleaking strategy specifically for tumor types overexpressing claudins, which are not CPE receptors such as Cldn1 by design and use of CPE mutants: *In vitro* and *in vivo*, tumor growth of PTC (K1 cells), overexpressing mainly Cldn1, but not Cldn3 and Cldn4, was efficiently reduced by Cldn1‐binding CPE‐variant S231R/S313H (CPE‐Mut3), but not by CPEwt. In addition, we show that CPE variants suppress growth of NSCLC tumors in a claudin‐directed manner.

## 2 Materials and methods

### 2.1 Plasmids

Plasmid encoding pGEX‐4T‐CPE194‐319 carrying cCPE with N‐terminal GST‐fusions was described (Veshnyakova *et al.*, 2012b). Single or multiple substitutions (N218Q, S231R, K283R, S305P, Y306A, Y306W, S307R, S313H, and L315A) were generated by site‐directed mutagenesis of pGEX‐4T1‐CPE194‐319, as described earlier (Winkler *et al.*, 2009).

For expression as N‐terminal 6xHis‐fusion, CPE cDNA was subcloned from vector pCpG‐optCPE (Walther *et al.*, 2012) as described earlier (Eichner *et al.*, 2017). Site‐directed mutagenesis to generate full lengths CPE‐Y306A/L315A (negative control), CPE‐S313H (Mut1), CPE‐S231R (Mut2), CPE‐S231R/S313H (Mut3), CPE‐S305P/S307R/S313H (Mut4), CPE‐S231R/Y306W/S313H (Mut5), CPE‐N218Q/Y306W/S313H (Mut6), CPE‐N218Q/S231R/S313H (Mut7), CPE‐N218Q/S231R/Y306W/S313H (Mut8), and CPE‐Y306W/S313H (Mut9) was performed as described (Winkler *et al.*, 2009). Plasmids based on pEYFP‐N1 encoding human Cldn1‐YFP, Cldn2‐YFP, Cldn5‐YFP, Cldn6‐YFP, Cldn7‐YFP, murine Cldn3‐YFP, as well as plasmid pEGFP‐Cld4 encoding human Cldn4 with N‐terminal GFP (Protze *et al.*, 2015) and plasmid encoding human Cldn1 with N‐terminal FLAG (Veshnyakova *et al.*, 2012a) have been described previously.

### 2.2 Expression and purification of cCPE and CPE constructs

GST‐cCPE constructs were expressed and purified as described (Protze *et al.*, 2015). Labeling according to Table S1.

6xHis‐CPE was expressed in *Escherichia coli* TOP10 (Thermo Fisher Scientific, Waltham, MA, USA) and purified from lysates using Ni‐NTA‐Agarose (Qiagen GmbH, Hilden, Germany), as described earlier (Eichner *et al.*, 2017). For negative control, preparations from bacteria transformed with vector containing CPE insert in antisense – and thus not expressing any CPE – were carried out in parallel, as described earlier (Eichner *et al.*, 2017).

### 2.3 Cell culture

All cell lines were purchased from Sigma‐Aldrich (Taufkirchen, Germany). Native HEK293 (HEK) cells and HEK cells, stably transfected with Cldn1‐7, were cultured as described elsewhere (Protze *et al.*, 2015). PC‐9 and Nthy‐ori 3‐1 cells were cultivated in RPMI (10% FBS; 1% penicillin/streptomycin, P/S), K1 – in DMEM (10% FBS; 1% P/S), and SK‐Mes‐1 – in MEM (10% FBS; 1% P/S, 1% nonessential amino acids) and were grown in a 5% CO_2_ atmosphere at 37 °C.

### 2.4 CPE transfection

When cells seeded on poly‐l‐lysine (Sigma‐Aldrich, St. Louis, MO, USA) precoated 96‐well plates reached 80% confluency, they were transiently transfected with 0.1 µg DNA of CPE constructs and vector with antisense CPE sequence (as negative control) using ViaFect (Promega, Madison, WI, USA).

### 2.5 MTT cytotoxicity assay

For MTT ((3‐4,5‐dimethylthiazol‐2‐yl)‐2,5‐diphenyltetrazolium bromide; Sigma‐Aldrich, USA) cytotoxicity assay cells were seeded 80% confluent on 96‐well plates (Sigma‐Aldrich, USA) precoated with poly‐l‐lysine. On next day, cells were preincubated at 37 °C, 5% CO_2_ for 1 or 24 h, with variable dilutions of cCPE or CPE preparations or 0.01% (v/v) Triton X‐100 (background correction) before medium was replaced by medium without phenol red containing 1.25 mm MTT. Two hours later, cells were treated with 5% (v/v) Triton X‐100 in 2‐propanol for 20 min and absorption was measured at 560 nm.

### 2.6 Cellular binding assay

Native claudin‐negative HEK cells are a suitable model to test specific targeting of single claudins, when those are expressed exogenously (Protze *et al.*, 2015). Two days after transient transfection or 1 day after plating of stable lines, HEK cells expressing one of the tested claudins (Cldn1‐7) were incubated with 0.5 µg·mL^−1^ GST‐cCPE constructs (30 min, 37 °C) in 24‐well plates (Sigma‐Aldrich, USA). Amount of bound cCPE was detected as described elsewhere (Protze *et al.*, 2015).

### 2.7 Cell lysis and western blotting

K1, Nthy‐ori 3‐1, PC‐9, and SK‐Mes‐1 cells were seeded in 25 cm^2^ cell culture flasks (Greiner Bio One, Kremsmuenster, Austria). After reaching confluency, cells were washed and harvested, and extracted membrane protein fraction was analyzed as described elsewhere (Veshnyakova *et al.*, 2012a). Proteins were detected using specific antibodies against Cldn1‐9 or with anti‐β‐actin (Thermo Fisher Scientific), respectively, and visualized by luminescence imaging (Lumi‐Imager, Roche, NY, USA).

### 2.8 Structural bioinformatics and molecular modeling

Homology models of claudin–cCPE complexes were built based on Cldn4–cCPEwt complex (PDB ID: http://www.rcsb.org/pdb/search/structidSearch.do?structureId=5B2G, Shinoda *et al.*, 2016). All manipulations, optimizations of models, were handled as described elsewhere (Neuhaus *et al.*, 2018).

### 2.9 *In vivo* CPE application

For cell line‐derived subcutaneous xenotransplant (CDX) tumor models, 5 × 10^5^ PC‐9 cells or 1 × 10^6^ K1 cells were injected subcutaneously into female NOG mice (*n* = 5 animals per group). Tumor volume (TV, calculated according to formula 0.5 × length × width^2^) and body weight were determined at least twice weekly. When tumors reached mean volume of 0.14–0.17 cm^3^, recombinant CPE was injected intratumorally in anesthetized animals. In total, 20 μg of recombinant CPE was applied by 10 injections (daily dose of 0.04 μg CPE·μL^−1^ 0.9% NaCl). During treatment, TV was measured at days 11, 14, 18, and 21 after injection of PC‐9 cells or at days 18, 21, 25, and 28 after injection of K1 cells. Animals were sacrificed for tumor removal and further analysis. All experiments were performed at EPO GmbH, Berlin, Germany.

### 2.10 Solid tumor tissue dissection

Shock frozen tumor tissues were fixed with Tissue‐Tek Medium (Sakura, Staufen, Germany) and dissected with cryostat into cryosections each of 10 or 18 μm thickness, which were transferred onto cover slides.

### 2.11 Hematoxylin and eosin staining

For morphological evaluation of 18 µm thick tissue sections, hematoxylin and eosin (HE) staining was performed. Staining procedures were performed by standard protocols (Fischer *et al.*, 2008). All samples were digitally scanned and viewed using Aperio ScanScope AT2 system (Leica Biosystems, Wetzlar, Germany) and qupath software (Bankhead *et al.*, 2017). Necrotic areas were calculated by determination of the whole tissue area vs necrotic tissue area using imagej (open source software, https://imagej.net/) in triplicates of two samples per group. Necrotic area in % related to the whole tissue area is shown.

### 2.12 Immunohistochemistry of human thyroid samples

Thyroid samples were obtained from patients undergoing thyroid surgery for PTC. For Cldn1 staining, paraffin‐embedded tissue sections of 10 PTC were studied. Classification of thyroid nodules was performed by pathologists according to World Health Organization criteria. Cldn1 staining with anti‐Cldn1 antibody was performed as described previously (Zwanziger *et al.*, 2015). All samples were digitally scanned and viewed using Aperio ScanScope AT2 system and qupath software.

### 2.13 Statistics

Data are expressed as mean ± SEM. Statistical analyses were performed with graphpad prism version 5.0 (San Diego, CA, USA) using one‐way or two‐way ANOVA and Bonferroni’s adjustment for multiple comparisons.

## 3 Results

### 3.1 Generation of cCPE variant with improved Cldn1 binding

We first aimed to create variants of the nontoxic claudin‐binding domain of CPE (cCPE) with increased Cldn1 binding. Mutation S313H in cCPE, was previously shown to improve Cldn1 binding and enable Cldn5 binding (Protze *et al.*, 2015; Takahashi *et al.*, 2012).

**Figure 1:**
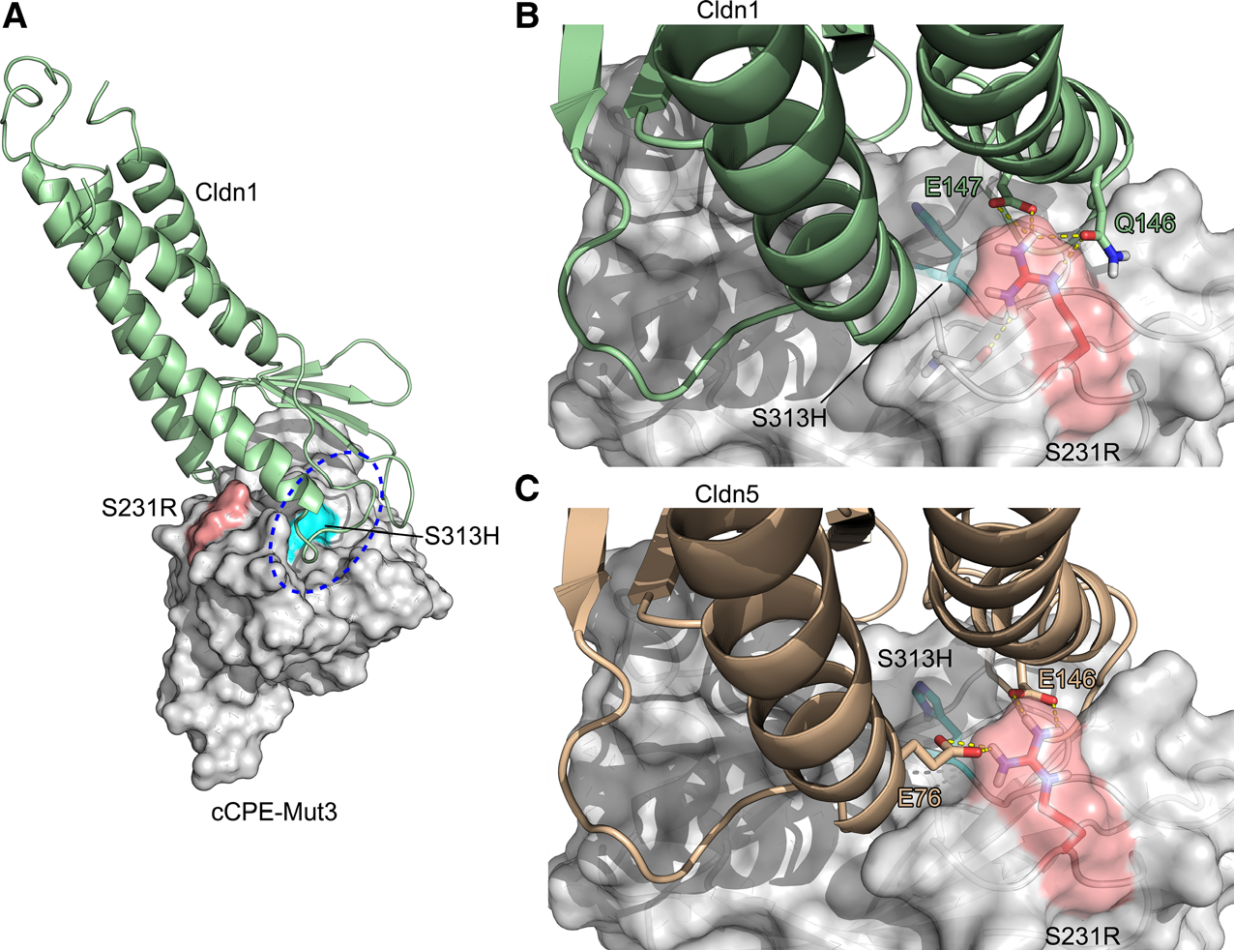
Predicted interaction of cCPE‐Mut3 with Cldn1 and Cldn5. (A) Extracellular domains (ECL1 and ECL2) of Cldn1 (green) interact with CPE residues, both within ECL2‐binding pocket (blue dashed line, Protze et al., 2015) and outside of it. Modeling of Cldn1‐CPE interaction interface (based on Cldn4‐cCPEwt complex, PDB ID: http://www.rcsb.org/pdb/search/structidSearch.do?structureId=5B2G, Shinoda et al., 2016) suggested the highlighted mutations (Mut1, blue; Mut2, red) on the cCPE surface (gray) to improve binding. (B) Close up view of modeled interaction between Cldn1 and cCPE‐Mut3. (C) Close up view of modeled interaction between Cldn5 and cCPE‐Mut3.

To further improve Cldn1 and Cldn5 binding of cCPE‐Mut1 (cCPE‐S313H), we generated models of claudin–cCPE‐S313H interaction (Fig. 1A–C). This pointed us to substitution of S231 to Arg to potentially enhance Cldn1‐ (Fig. 1B) and Cldn5 binding (Fig. 1C). According to our models, S231R in cCPE (cCPE‐Mut2) should allow formation of hydrogen bonds with Q146 and E147 in Cldn1 improving Cldn1‐binding properties of cCPE (Fig. 1B) and with E76 and E146 enabling interaction with Cldn5 (Fig. 1C). Furthermore, it potentially enhances binding to Cldn2 by interacting with D76 which corresponds to E76 in Cldn5.

**Figure 2:**
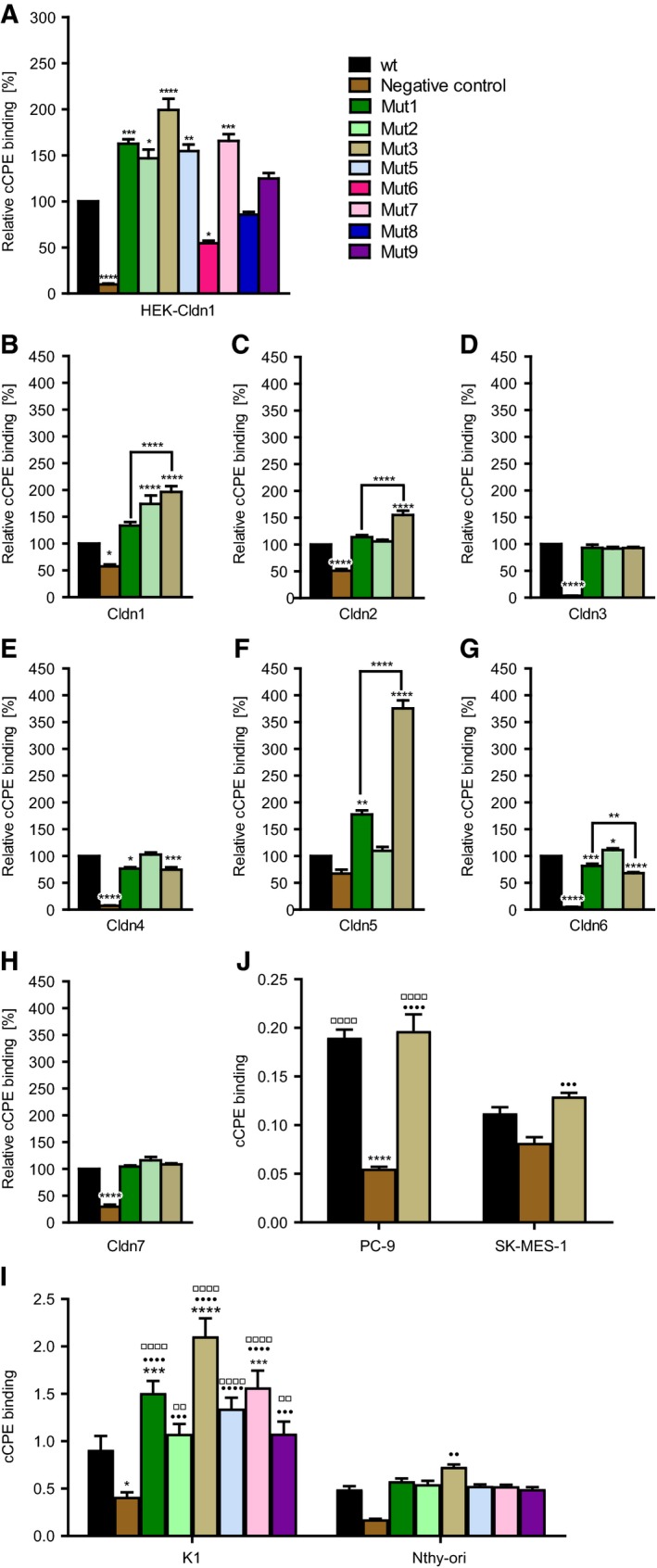
Double mutation S231R/S313H (Mut3) increases claudin binding of cCPE. (A) Binding assays with transiently Cldn1‐transfected HEK cells revealed that cCPE‐Mut3 is the strongest interaction partner of Cldn1 in comparison with the other cCPE variants. Binding assays with HEK cells stably transfected with Cldn1‐YFP (B), Cldn2‐YFP (C) Cldn3‐YFP (D), Cldn4‐GFP (E), Cldn5‐YFP (F), Cldn6‐YFP (G), or Cldn7‐YFP (H) showed that cCPE‐Mut3 binds stronger to Cldn1, Cldn2, and Cldn5, then cCPEwt. Similarly to Cldn1‐transfected HEK cells, binding assays with PTC model cell line K1 cells and Nthy‐ori 3‐1 (Nthy‐ori) thyroid follicular epithelial cells (I) showed strongest binding for cCPE‐Mut3. cCPE bound stronger to K1 than to Nthy‐ori cells. (J) cCPE‐Mut3 and cCPEwt bound stronger than cCPE‐negative control to PC‐9 and SK‐MES‐1 cells. cCPE‐Mut3 and cCPEwt bound stronger to PC‐9 than to SK‐MES‐1 cells. Cells were incubated for 30 min with different GST‐cCPE constructs (0.5 µg·mL^−1^), washed, and the bound cCPE was detected via anti‐GST‐R‐phycoerythrin antibodies. The signal was normalized to cell number (A, I, J) or claudin expression level (B‐H, YFP‐ or GFP signal), and the binding of cCPE mutants relative to cCPEwt was calculated. One‐way ANOVA, * vs cCPEwt or as indicated, ● vs cCPE‐negative control, □ vs treatment in low claudin‐expressing cells (K1 vs Nthy‐ori and PC‐9 vs SK‐MES‐1, respectively). *n* ≥ 3. *, *P* < 0.05; **, □□, *P* < 0.01; ***, ●●● *P* < 0.001; ****, ●●●●, □□□□, *P* < 0.0001.

In sum, molecular models suggests that mutation S231R increases the interaction with Cldn1 and Cldn5. Mutants that previously showed effects on interaction with Cldn1 and Cldn5 (N218Q, Neuhaus *et al.*, 2018; Y306W and S313H, Protze *et al.*, 2015) were here tested in the following combinations: The model‐guided recombinant cCPE variants Mut1 (cCPE‐S313H), Mut2 (cCPE‐S231R), Mut3 (cCPE‐S231R/S313H), Mut5 (S231R/Y306W/S313H), Mut6 (N218Q/Y306W/S313H), Mut7 (N218Q/S231R/S313H), Mut8 (N218Q/S231R/Y306W/S313H), Mut9 (cCPE‐Y306W/S313H), and negative control (cCPE‐Y306A/L315A) not binding to claudins (Protze *et al.*, 2015; Table S1) were expressed in *E. coli*, and purified as previously reported (Protze *et al.*, 2015; Veshnyakova *et al.*, 2012b). Binding of these cCPE variants toward Cldn1 was tested on HEK cells transiently transfected with Cldn1‐YFP (Fig. 2A). Single substitutions Mut1 and Mut2 significantly enhanced binding toward Cldn1 and boosted it further, when combined in double mutant Mut3. Moreover, combining single mutation Mut2 with other double mutations resulting in triple mutations cCPE‐Mut5 or cCPE‐Mut7 increased binding to Cldn1, too. Four substitutions (cCPE‐Mut8) showed intermediate effect. Mut6 was employed as additional negative control, since it is known to decrease Cldn1 binding, but to increase Cldn5 binding of cCPE (Neuhaus *et al.*, 2018). In summary, the data showed that cCPE‐Mut3 has the best binding properties toward Cldn1.

To test whether cCPE candidate mutations (Mut3 and related Mut1/Mut2, Table S1) influence binding properties toward other claudins, HEK cells stably expressing YFP‐tagged single claudins (Cldn1, Cldn2, Cldn3, Cldn5, Cldn6, or Cldn7) or GFP‐tagged Cldn4 were employed in cellular binding assay. The YFP‐ or GFP‐tag located intracellularly did not influence the extracellular interaction between claudins and cCPE variants (Veshnyakova *et al.*, 2012b; Winkler *et al.*, 2009). As predicted by modeling, compared to cCPEwt, cCPE‐Mut3 bound significantly stronger to Cldn1, Cldn2, and Cldn5 (Fig. 2B,C,F), but weaker to Cldn4 and Cldn6 (Fig. 2E,G). There was no change in binding to Cldn3 (Fig. 2D).

### 3.2 CPE‐Mut3 enables Cldn1‐directed cytotoxicity

**Figure 3:**
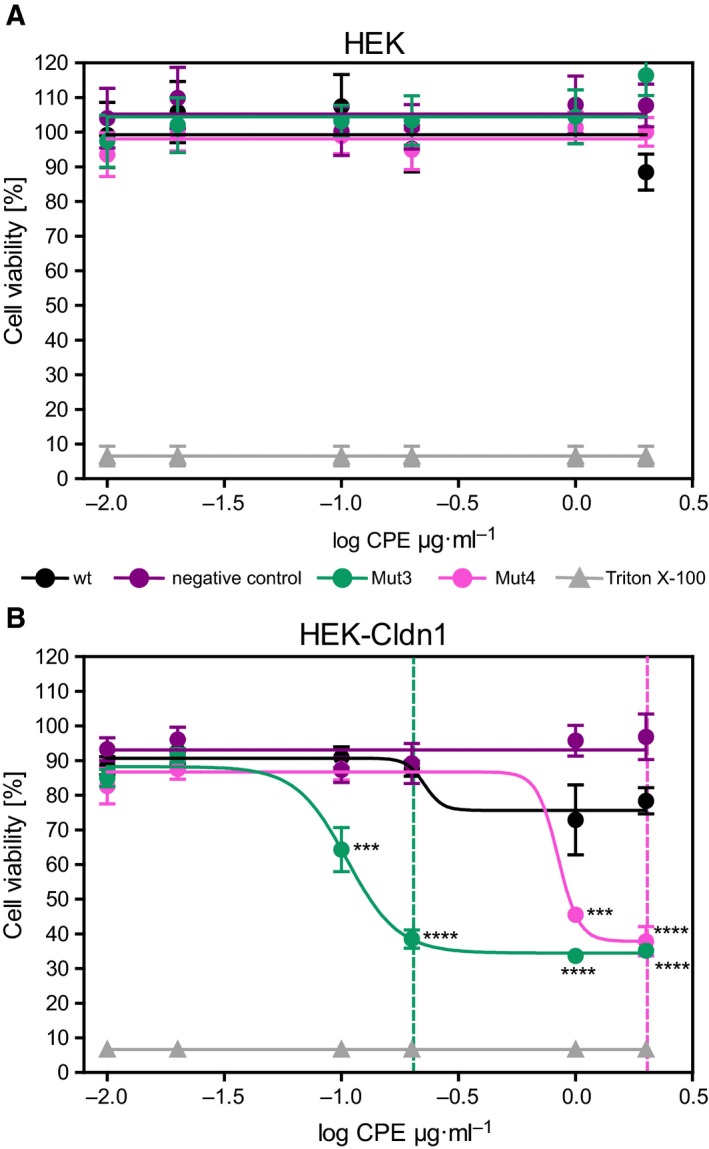
Cytotoxicity of CPE variants toward Cldn1‐expressing HEK cells depends on their Cldn1‐binding ability. Nontransfected (A) or stably Cldn1‐FLAG‐transfected HEK cells (B) were exposed to full length CPE variants applied at different concentrations (0.02–2.0 µg·mL^−1^) for 24 h, 37 °C. MTT cytotoxicity assay revealed that only Cldn1‐transfected cells were sensitive to CPE variants. CPE‐Mut3 and CPE‐Mut4 but not CPEwt or CPE‐negative control reduced cell viability considerably. Strikingly, CPE‐Mut3 was cytotoxic at much lower concentration (0.2 µg·mL^−1^, green dotted line) than CPE‐Mut4 (2.0 µg·mL^−1^ magenta dotted line). Growth medium with 0.1% Triton X‐100 was used as control for cell lysis. *n* = 3. Two‐way ANOVA with Bonferroni multiple comparisons. ***P < 0.001; ****P < 0.0001 significant vs wt (at the same concentration).

Next, substitutions Mut1 and Mut2 were introduced into His‐tagged full length CPE resulting in CPE‐Mut3 to investigate its Cldn1‐related cytotoxicity. For comparison, Mut4 (S305P/S307R/S313H), previously shown to increase binding of cCPE to Cldn1 and other claudins, similar as S313H (Protze *et al.*, 2015; Takahashi *et al.*, 2012), was also introduced into full length CPE. CPEwt, CPE‐Mut3, CPE‐Mut4, and CPE‐negative control (Y306A/L315A, not interacting with claudins, Eichner *et al.*, 2017) were applied to nontransfected and stably Cldn1‐FLAG‐transfected HEK cells. Nontransfected HEK cells were insensitive to any of the tested full length CPE variants (Fig. 3A). In contrast, Cldn1‐FLAG‐expressing HEK cells were highly sensitive to CPE‐Mut3, which strongly reduced cell viability already at low concentrations (0.2 µg·mL^−1^, Fig. 3B). The broad specificity binder CPE‐Mut4 had to be applied in 10‐fold higher concentrations – 2 µg·mL^−1^ to be as cytotoxic as CPE‐Mut3 (Fig. 3B). CPEwt or CPE‐negative control did not reduce cell viability for Cldn1‐FLAG‐expressing HEK cells significantly.

Thus, CPE‐Mut3, but not other CPE variants, enables efficient Cldn1‐directed cytotoxicity. Similarly, structure‐based mutagenesis of CPE was used to enable Cldn5‐directed cytotoxicity or to narrow down cytotoxicity toward Cldn4 (Fig. S1). Mechanistically, these CPE variants mediate cytotoxicity as CPEwt by induction of Ca^2+^‐influx (Fig. S2).

### 3.3 Expression of endogenous claudins in PTC and NSCLC cell models

**Figure 4:**
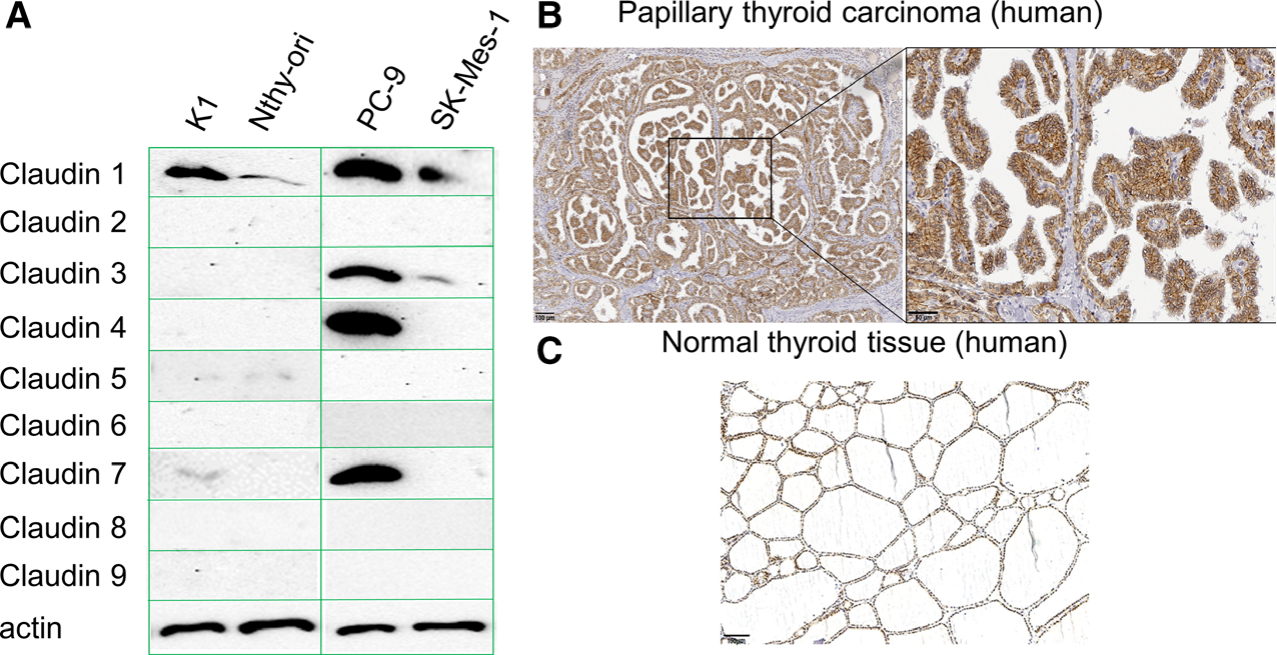
Claudin expression profile of thyroid and lung cancer cells. (A) The expression of endogenous Cldn1 to Cldn9 (20–22 kDa) in human PTC (K1 cells) and thyroid follicular epithelial Nthy‐ori 3‐1 cells (Nthy‐ori) as well as in human lung AC (PC‐9 cells) and squamous‐cell carcinoma (SK‐Mes1 cells) was analyzed by western blot. Equal loading of each lane was verified by β‐actin detection. *n* = 3. (B) Immunohistochemical analysis of Cldn1 in PTC. Strong Cldn1 plasma membrane staining in PTC. Representative photomicrographs are shown. *n* = 10. Scale bar 100 µm (left) and 50 µm (right). (C) Immunohistochemical analysis of Cldn1 in normal thyroid tissue. Representative photomicrograph is shown. *n* = 3. Scale bar 100 µm.

We aimed to test cytotoxicity of Cldn1‐binding CPE variants on thyroid and lung cancer cell lines, such as K1 cells (PTC) and Nthy‐ori 3‐1 cells (transformed from normal human thyroid follicular epithelial cells), PC‐9 (lung adenocarcinoma), and SK‐Mes‐1 (lung squamous‐cell carcinoma). Expression of Cldn1, as well as of other claudins (Cldn2 to Cldn9), in these cell lines was analyzed by western blot (Fig. 4A). For K1 and Nthy‐ori 3‐1 cells, expression analysis (Fig. 4A) revealed predominant Cldn1 expression with higher Cldn1 expression in K1 cells. This claudin expression matched to the one in human PTC tissue for which pronounced plasma membrane staining of Cldn1 was observed (Fig. 4B). In normal human thyroid tissue, Cldn1 was moderately expressed in plasma membrane and cytoplasm (Fig. 4C; Zwanziger *et al.*, 2015). Tested lung cancer cell lines expressed not only Cldn1, but also Cldn3, Cldn4, and Cldn7 (PC‐9 cells) or Cldn3 (SK‐Mes‐1 cells).

Since Cldn1 expression in PC‐9 cells was higher than that of SK‐Mes‐1 cells, PC‐9 cells were used as model for NSCLC for further experiments. K1 cells were chosen as model for PTC and Nthy‐ori 3‐1 cells as thyroid follicular epithelial cell model.

### 3.4 CPE‐Mut3 reduces cell viability of thyroid cancer cells *in vitro*

First, we tested binding of nontoxic cCPE variants to K1 and Nthy‐ori 3‐1 cells expressing Cldn1. Similarly as found for HEK‐Cldn1 cells (Fig. 2A), cCPE‐Mut3 bound stronger than other cCPE variants to both cell lines (Fig. 2I). Furthermore, stronger binding of cCPE‐Mut3 (relative to cCPEwt) to K1 compared with Nthy‐ori 3‐1 cells correlated with higher Cldn1 expression in K1 cells (Fig. 4A). The data showed that Mut3 enables binding of cCPE to Cldn1‐expressing thyroid cancer cells.

**Figure 5:**
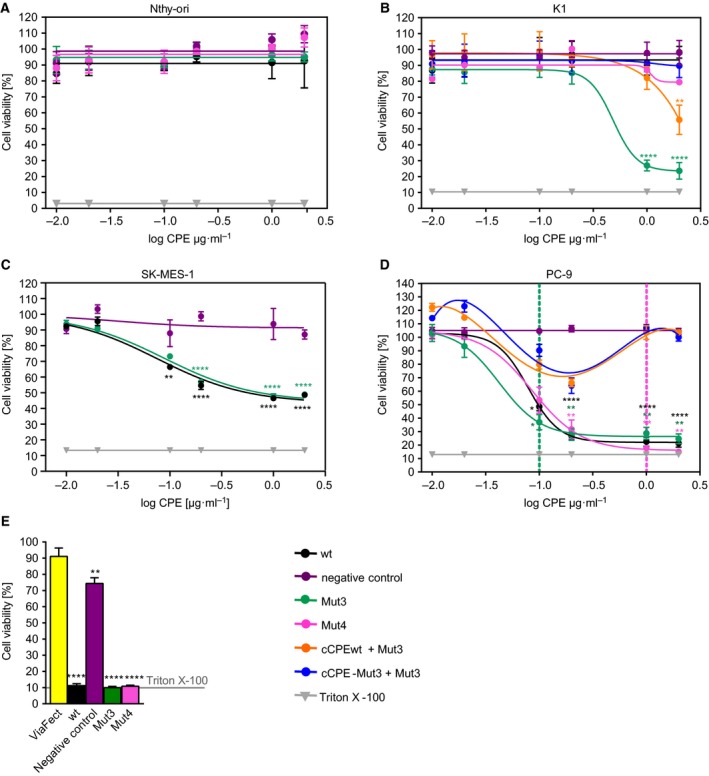
Claudin‐binding CPE variants are highly cytotoxic for K1 thyroid and PC‐9 lung carcinoma cells. Viability of Nthy‐ori 3‐1 cells (Nthy‐ori) (A) and K1 cells (B) 24 h after application of CPE variants at different concentrations. For Nthy‐ori 3‐1 cells weakly expressing Cldn1, none of the treatments reduced cell viability. For K1 cells strongly expressing Cldn1, CPE‐Mut3 drastically reduced cell viability. Cells were either incubated with CPEwt, CPE‐negative control, CPE‐Mut3, or CPE‐Mut4 or simultaneously treated with cCPEwt or cCPE‐Mut3 and CPE‐Mut3. Afterward, MTT assay was performed. In competition assay, simultaneous incubation with both nontoxic cCPE‐Mut3 and full length CPE‐Mut3 (blue line) protected K1 cells from CPE‐Mut3‐mediated cytotoxicity (green line). cCPEwt added together with CPE‐Mut3 (orange line) showed just a weak protective effect, and CPE‐negative control (purple line) and CPEwt (black line) did not affect K1 cells. (C) SK‐MES‐1 cells were incubated for 1 h with CPE variants (wt, Mut3 and negative control) at different concentrations followed by MTT assay. CPEwt and CPE‐Mut3 reduced cell viability to ~ 50%. (D) PC‐9 cells expressing multiple claudins were efficiently attacked by CPEwt, CPE‐Mut3, and CPE‐Mut4. Cells were incubated for 1 h with CPE variants (wt, Mut3, Mut4, and negative control) at different concentrations followed by MTT assay. Where indicated, cells were preincubated for 1 h with nontoxic cCPE (wt or Mut3) before CPE‐Mut3 application. Partial inhibition effect at concentrations lower than 0.2 µg·mL^−1^ and full inhibition at higher concentrations (blue, orange lines) indicated that low cCPE concentration was not sufficient to occupy all CPE receptors. (E) PC‐9 cells were transfected with DNA (0.1 µg) of CPE constructs, and 24 h later, MTT cytotoxicity assay was performed. All CPE constructs, except for CPE‐negative control, were toxic similarly to 0.1% Triton X‐100. Application of growth medium with 0.1% Triton X‐100 was used as control for cell lysis in (A–E). *n* = 3–11 (K1 cells + Mut4 *n* = 1), one‐way ANOVA with Bonferroni multiple comparisons significant vs CPE‐negative control (at the same concentration) **P < 0.01; ***P < 0.001; ****P < 0.0001 (A–D), or one‐way ANOVA with Bonferroni multiple comparisons significant vs ViaFect, (*n* = 4) *P < 0.05; **P < 0.01; ***P < 0.001 (E).

Consequently, cytotoxicity of full length CPE‐Mut3 was tested on Nthy‐ori 3‐1 and K1 cells using MTT assays. After 24‐h incubation with CPEwt, CPE‐negative control, CPE‐Mut3, and CPE‐Mut4, viability of Nthy‐ori 3‐1 cells was not affected (Fig. 5A). In contrast, incubation of K1 cells with the Cldn1‐binders CPE‐Mut3 and CPE‐Mut4 (Fig. 5B, green and pink) reduced cell viability. At highest concentration, CPE‐Mut3 reduced K1 cell viability remarkably to about 20% of negative control levels, whereas CPE‐Mut4 decreased it to about 80% of control levels, only. The CPE‐negative control and CPEwt (Fig. 5B, black) did not have any toxic effect on K1 cells.

To further verify that CPE‐mediated cytotoxicity depends on presence of claudins on surface of K1 cells, K1 cells were incubated simultaneously with both noncytotoxic cCPE (wt or Mut3) and cytotoxic full length CPE‐Mut3 in same molar concentrations. cCPE‐Mut3 bound to Cldn1 on the surface of K1 cells and thereby protected them completely from cytotoxic effect of full length CPE‐Mut3 (Fig. 5B, blue). In contrast, due to poor interaction of CPEwt with Cldn1, it could not fully protect K1 cells from CPE‐Mut3‐mediated cytotoxicity (Fig. 5B, orange).

Thus, unlike CPEwt and the broad specificity binder CPE‐Mut4, the designed CPE‐Mut3 strongly reduced cell viability selectively in K1 cells overexpressing Cldn1 (*in vitro* PTC model). The insensitivity of Nthy‐ori 3‐1 cells to CPE‐Mut3 is likely due to their low Cldn1 expression and suggests that CPE‐Mut3‐mediated cytotoxicity could potentially be restricted to Cldn1‐overexpressing malignant PTC.

### 3.5 CPE variants are cytotoxic for PC‐9 lung cancer cells

Similar as for thyroid follicular epithelia and PTC cells, we tested binding of nontoxic cCPE variants to PC‐9 and SK‐MES‐1 lung cancer cells. cCPEwt and cCPE‐Mut3 bound much stronger to PC‐9 cells than the cCPE‐negative control (Fig. 2J). cCPEwt and cCPE‐Mut3 bound stronger to PC‐9 cells than to SK‐MES‐1 cells. Nevertheless, cCPE‐Mut3 bound stronger than cCPEwt and the latter stronger than the cCPE‐negative control to SK‐MES‐1 cells (Fig. 2J). cCPE binding to the different cell lines correlates to their respective claudin expression profile and level (Fig. 4A).

To investigate the cytotoxic effect of CPE variants on lung cancer cells, SK‐MES‐1 and PC‐9 cells were incubated with CPE variants for 1 h. In contrast to the CPE‐negative control, CPEwt and Mut3 decreased cell viability of SK‐MES‐1 cells at concentrations ≥ 0.1 µg·mL^−1^ (Fig. 5C). For PC‐9 cells expressing multiple claudins (Fig. 4A) and showing strong cCPE binding, incubation with CPEwt, Mut3, and Mut4 strongly decreased cell viability at concentrations ≥ 0.1 µg·mL^−1^ (Fig. 5D, green dotted line). At concentrations ≥ 1 µg·mL^−1^, these CPE variants reduced cell viability similarly to the level of cell lysis positive control (0.1% Triton X‐100, about 20%, Fig. 5D, magenta dotted line). As expected, CPE‐negative control did not reduce cell viability even at high concentrations. The stronger CPE sensitivity of PC‐9 compared to SK‐MES‐1 cells correlated to their claudin expression profiles and levels (Fig. 4A).

Competition cytotoxicity assay with nontoxic cCPE variants demonstrated that full length CPE‐Mut3 requires claudin molecules on the plasma membrane. PC‐9 cells were preincubated with cCPEwt or cCPE‐Mut3 for 1 h before 1‐h incubation with the same molar concentration of full length CPE‐Mut3. Preincubation of PC‐9 cells with cCPEwt or cCPE‐Mut3 led to competition for claudin binding and at high concentrations strongly inhibited reduction in cell viability mediated by CPE‐Mut3 (Fig. 5C, blue and orange), indicating that protective effect of cCPE depends on its concentration. Thus, CPE‐Mut3‐mediated cytotoxicity depends on claudins on the surface of PC‐9 cells. The data showed that claudin‐binding CPE variants can efficiently attack lung cancer cells expressing multiple claudins.

### 3.6 CPE application via DNA transfection

As an alternative application route, CPE transfection (Walther *et al.*, 2012) was used for comparison. 0.1 µg DNA of CPE constructs or controls were transiently transfected into PC‐9 cells, and 24 h later, cytotoxicity assay was performed. MTT assays revealed similar results as with recombinant CPE: CPEwt, CPE‐Mut3, and CPE‐Mut4, but not the nonbinding CPE‐negative control strongly reduced cell viability (Fig. 5E). Our data support the rigorous claudin dependency of CPE toxicity independently of the route of application as shown before (Walther *et al.*, 2012).

Thus, two independent approaches of CPE application (recombinant protein and DNA transfection) demonstrated for PC‐9 cells (a) claudin‐dependent cytotoxicity of CPE and (b) that all claudin‐sensitive CPE variants (CPEwt, CPE‐Mut3 and CPE‐Mut4) reduced cell viability. The latter can be explained by the fact that PC‐9 cells do not only express Cldn1, but also Cldn3, Cldn4, and Cldn7 (Fig. 4A), which strongly interact with CPEwt and CPE‐Mut4.

### 3.7 CPE treatment reduces tumor growth of thyroid and lung cancer *in vivo*

Finally, we tested *in vivo* the effect of CPE variants on tumor growth on CDX models. For establishment of subcutaneous tumors, K1 or PC‐9 cells were injected into female NOG mice. When tumors reached a mean volume of 0.14–0.17 cm^3^, 10 intratumoral injections of recombinant CPE (wt or ‐Mut3) or of vector control preparations were administrated within 10 days (single 2 µg injection daily).

**Figure 6:**
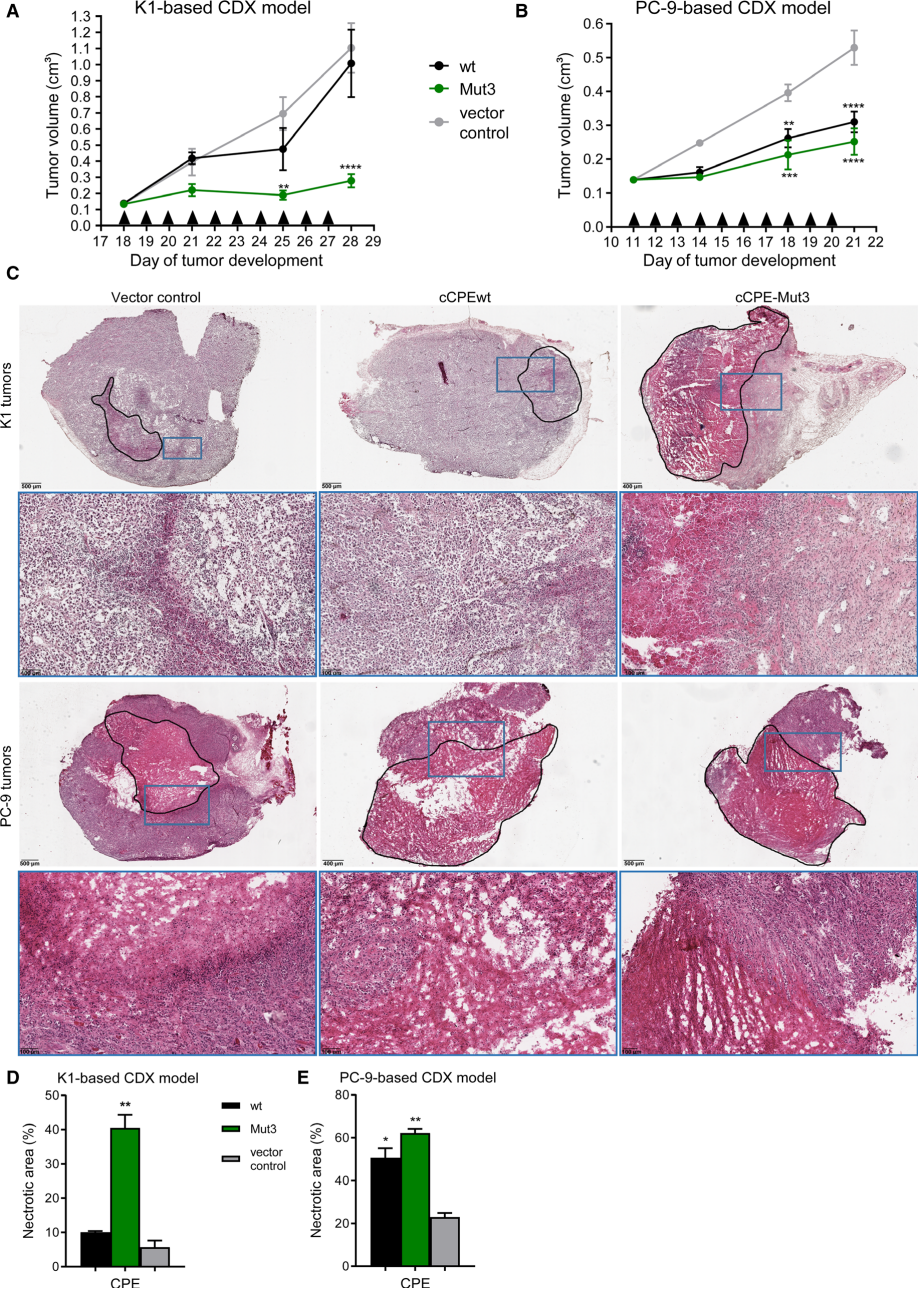
Effect of recombinant CPE treatment on tumor growth. K1 cells (A) or PC‐9 cells (B) were injected s.c. into female NOG mice. When tumors reached a mean volume of 0.14 cm^3^, animals were randomized into treatment groups and *in vivo* recombinant CPE intratumoral application was performed. Thus, CPEwt or CPE‐Mut3 was applied by 10 injections (arrow heads) (0.04 μg CPE·μL^−1^ 0.9% NaCl, daily). (A) K1‐TV in the group, treated with CPE‐Mut3, was 25% compared to those treated with vector control or CPEwt. CPEwt did not affect the K1 cells and therefore did not reduce the TV (i) due to the absence of Cldn3 and Cldn4 in these cells, the main receptors of CPEwt, and (ii) due to its inability to strongly interact with Cldn1. *n* = 4–5. (B) PC‐9‐ TV in the group, treated with CPEwt or CPE‐Mut3, was only 58% or 47%, respectively, of those treated with vector control. *n* = 5. (C) Histological analysis of HE‐stained K1 and PC‐9 xenograft tumors showing the different degrees of tumor necrosis. Necrotic areas are contoured with black line. Representative photomicrographs are shown. Lower panels display higher magnification of the blue boxes. (D) Quantification of necrotic area in K1 tumor. (E) Quantification of necrotic area in PC‐9 tumor. Quantification of necrotic area was performed with 2 tumors, each 3 sections, in total 6 areas per group. Statistical analysis was performed with two‐way ANOVA with Bonferroni multiple comparisons (A, B) and Kruskal–Wallis test with Dunn’s multiple comparison test (D, E) significant vs vector control (at the same concentration) **P < 0.01; ***P < 0.001; ****P < 0.0001.

In the PTC K1 xenograft model, during treatment by vector control and CPEwt the tumor was growing strongly to 1.1 ± 0.15 cm^3^ and to 1.0 ± 0.21 cm^3^, respectively. In contrast, for CPE‐Mut3‐treated animals, only minor tumor growth (to 0.28 ± 0.04 cm^3^) was observed. The study revealed no significant effect of CPEwt treatment, but a very efficient anti‐tumoral activity of CPE‐Mut3, reflected by reduced tumor growth (14% compared to control) (Fig. 6A). Hence, unlike CPEwt, the CPE variant with increased affinity to Cldn1 binds stronger to PTC cells. This binding is associated with more efficient tumor cell killing, resulting in tumor reduction.

In the PC‐9 xenograft model, measurements of TV on days 11, 14, 18, and 21 after beginning of CPE‐administration in different treatment groups revealed anti‐tumoral activity of both CPEwt and of CPE‐Mut3, reflected by respective TVs of 43% and 29% (compared to vector control treatment, Fig. 6B). Here, CPE‐Mut3 exerted only slightly improved anti‐tumoral effects than CPEwt. Thus, intratumoral CPE treatment is able to reduce TV in lung cancer PC‐9 CDX model effectively.

### 3.8 CPE treatment causes necrosis in thyroid and lung tumor

Intratumoral injection of CPE variants reduced significantly TV of K1 and PC‐9 xenograft models. HE staining revealed a small amount of 5–10% of necrotic areas of K1 tumors treated with vector control and CPEwt, whereas CPE‐Mut3 application increased necrotic areas of K1 tumors to approximately 40% (Fig. 6C,D). In PC‐9 tumors, necrotic areas of vector control treated mice were approximately 23% and elevated to 51% and 63% by CPEwt and CPE‐Mut3 application, respectively (Fig. 6C,E). Taken together, the *in vivo* data indicate that CPE treatment reduces tumor growth by induction of necrosis.

In thyroid and lung tumor xenograft models, immunostaining of claudins and ZO1 did not reveal a junctional localization, pointing to absence of intact TJ. In thyroid xenograft model, the analysis showed strongest CPE and weakest Cldn1 signals in CPE‐Mut3‐treated samples (Fig. S3). For lung xenograft model CPEwt and CPE‐Mut3 treatment, the immunostainings did not reveal a clear change in expression and distribution of Cldn3, Cldn4, and Cldn7 (Fig. S3).

## 4 Discussion

In this study, we used structure‐guided mutagenesis of CPE to enable for the first time cytotoxic targeting of tumors expressing Cldn1 or Cldn5 to which CPEwt does not bind considerably. As a proof of principle, we showed *in vitro* and *in vivo* that claudin‐binding properties of CPE can be shifted (a) to expand use of CPE to targeting of Cldn1‐expressing thyroid tumors and (b) to potentially reduce CPE side effects by matching the binding properties of CPE to the claudin expression profile of particular carcinoma types.

This study aimed to overcome the restriction of the promising CPE treatment to tumors expressing only receptors for CPEwt (Cldn3, Cldn 4, Cldn6, Ben‐David *et al.*, 2013; Kominsky *et al.*, 2007; Kominsky *et al.*, 2004; Liang *et al.*, 2017; Michl *et al.*, 2001; Santin *et al.*, 2005; Walther *et al.*, 2012). We adapted the CPE binding properties to other claudins according to the expression profile of different carcinomas. In particular, binding to Cldn1 expressed in NSCLC (Jung *et al.*, 2009; Sun *et al.*, 2016) and thyroid cancer (Zwanziger *et al.*, 2015) was intended. The role of Cldn1 in follicular type of thyroid cancer was well described (Zwanziger *et al.*, 2015). However, for PTC, the most common type of thyroid cancer, the Cldn1 expression, and its role in pathogeny are currently poorly characterized. In this study, we concentrated on PTC since this is the most common type of all thyroid cancers.

First, we verified if model‐guided variants of cCPE – the nontoxic claudin‐binding domain of CPE – were able to interact with Cldn1 to Cldn7. cCPE‐Mut3 with increased binding to Cldn1 was chosen as the most promising candidate for PTC cells’ eradication. Its additional ability to interact with Cldn5 is of advantage for potential NSCLC treatment, since NSCLC is characterized by upregulation of several claudins including both Cldn1 and Cldn5 (Paschoud *et al.*, 2007).

Consequently, Mut3 and, as comparison, Mut4 – which was previously shown to improve cCPE binding to Cldn1 and Cldn5 – were introduced into cytotoxic full length CPE and showed identical claudin specificity as corresponding cCPE variants. In contrast to CPEwt, CPE‐Mut3 and CPE‐Mut4 were cytotoxic for Cldn1‐overexpressing but not for native HEK cells. This finding demonstrated the ability of novel CPE variants to specifically target Cldn1‐expressing cells.

Furthermore, in contrast to CPEwt, CPE‐Mut3 was cytotoxic for K1 thyroid cancer cells expressing endogenous Cldn1. For PC‐9 lung cancer cells that express multiple claudins, apart from CPE‐Mut3 also CPEwt and CPE‐Mut4 mediated cytotoxicity. Finally, claudin‐specific cytotoxicity was confirmed *in vivo* on CDX models of PTC (K1 cells) and of NSCLC (PC‐9 cells). For the first time, CPE was used to successfully reduce growth of lung tumors (PC‐9 cells) and its Mut3 enabled efficient Cldn1‐directed reduction of PTC (K1 cells) growth.

Using the same approach of structure‐guided mutagenesis, we also generated CPE‐Mut9 and CPE‐Mut10 which enabled efficient Cldn5‐ and Cldn4‐directed cytotoxicity, respectively (Fig. S1). Thus, CPE variants with claudin‐binding properties matching to the claudin expression profile of the targeted tumor type have a potential to maximize tumor cell eradication and minimize side effects on normal claudin‐expressing cells.

In several studies, high claudin specificity of anti‐claudin antibodies (Cldn1, Yoda *et al.*, 2014; Cldn4, Silva *et al.*, 2017; Cldn5, Hashimoto *et al.*, 2017; and Cldn18.2, Singh *et al.*, 2017) was used for cancer cell targeting. However, recombinant CPE variants as anti‐tumoral tools have several advantages over anti‐claudin antibodies’ application: (a) cost‐effective bacterial production of CPE, (b) ability to adjust CPE, by directed structural modification to target several claudin subtypes and thereby target higher number of claudin molecules, causing faster and more severe eradicating effect, and (c) in CPE, the claudin‐targeting and effector (toxic) domains are already natively combined, whereas for other immunotoxins and antibodies targeting and toxicity have to be mediated independently.

Moreover, CPE can be applied by cost‐ and labor‐effective nonviral *in vivo* gene transfer (Walther *et al.*, 2012). This results in CPE production in target cells, causing direct cell killing further enhanced by a toxin‐mediated bystander effect. The claudin specificity and dependency of the cytotoxicity mediated by CPE expressed after gene transfer in mammalian cells was demonstrated previously (Pahle *et al.*, 2017; Walther *et al.*, 2012). In the current study, gene transfer of CPE variants mediated a similar toxicity on the NSCLC model *in vitro* (PC‐9 cells) as the recombinant protein (Fig. 5D,E). Thus, the suicide gene transfer approach can be broadened to CPE variants. However, here we applied recombinant CPE for *in vivo* experiments, since this enabled better control of CPE concentration.

Intratumoral *in vivo* application of recombinant CPE did not induce general toxin‐associated side effects in PC‐9 and K1 xenograft models bearing mice, supporting its great therapeutic potential (Kominsky *et al.*, 2007). However, use of CPE for tumor therapy is restricted by the fact that CPE‐binding claudins are expressed in numerous tissues and, thereby, there is risk of unwanted cytotoxic damage of normal cells. Nevertheless, most tumor cells are assumed to be more sensitive to CPE than differentiated epithelial cells due to (a) overexpression and (b) higher accessibility of claudins caused by extrajunctional mislocalization of the molecules (Corsini *et al.*, 2018; Eichner *et al.*, 2017) (Fig. S3). In contrast, for example, in colonocytes, claudin accessibility, and in turn CPE‐mediated tissue damage, is strongly limited (Eichner *et al.*, 2017). However, better restriction of CPE‐mediated cytotoxicity to tumors might be achieved by use of CPE variants with a claudin‐subtype specificity optimized to match the claudin (over‐) expression profile of defined carcinomas. Nevertheless, extent of side effects due to CPE binding to claudins outside tumors has to be investigated in future *in vivo* studies with CPE variants.

A potential immune response against CPE could be addressed, for instance, (a) by subsequent use of cytotoxic cCPE‐PSIF (protein synthesis inhibitory factor) fusion proteins (Ebihara *et al.*, 2006) or (b) structure‐based modification of surface residues of CPE outside of the claudin‐binding region.

## 5 Conclusions

In summary, we showed that CPE can be modified by structure‐guided mutagenesis to match the binding properties of CPE to the claudin expression profile of particular carcinoma types. We thereby enabled, as proof of principle, Cldn1‐directed cytotoxic targeting of thyroid tumors. The results suggest use of CPE‐based biologics for eradication of claudin‐overexpressing cancers in a novel cross‐entity approach.

## Conflict of interest

The authors declare no conflict of interest.

## Author contributions

AP, ME, L‐SB, and JoP performed experiments. JP and GK conceived the study. AP, DZ, WW, ST, KWS, and JP interpreted data. AP, DZ, L‐SB, JP, and GK wrote manuscript. JP, WW, and GK supervised the study. WW, KWS, and DF approved final version. All authors commented on manuscript.

## Ethics approval and consent to participate

For use of human material, this study adhered to Declaration of Helsinki. Ethics approval for research was obtained from Ethics Committee of the University of Duisburg‐Essen, University Hospital Essen (approval number 12‐5133‐BO).

All animal experimental procedures are performed in accordance with the in‐house guidelines of the Institutional Animal Care and in accordance with the German Animal Protection Law and approved by the local responsible and official authorities, State Office of Health and Social Affairs (LaGeSo Berlin, Germany; Approval number A0452/08).

## Supporting information


**Fig. S1.** Shift of claudin‐subtype‐directed cytotoxicity of CPE by structure‐guided mutation of CPE.
**Fig. S2.** Claudin‐subtype‐directed cytotoxicity of CPE variants is mediated by induction of Ca^2+^ influx.
**Fig. S3.** Visualization of CPE and claudins in thyroid and lung xenograft models after recombinant CPE treatment.
**Table S1.** cCPE or CPE variants and claudin‐binding characteristics.

## References

[mol212615-bib-0001] Bankhead P , Loughrey MB , Fernandez JA , Dombrowski Y , McArt DG , Dunne PD , McQuaid S , Gray RT , Murray LJ , Coleman HG *et al* (2017) QuPath: open source software for digital pathology image analysis. Sci Rep 7, 16878. 29203879 10.1038/s41598-017-17204-5 PMC5715110

[mol212615-bib-0002] Ben‐David U , Nudel N and Benvenisty N (2013) Immunologic and chemical targeting of the tight‐junction protein claudin‐6 eliminates tumorigenic human pluripotent stem cells. Nat Commun 4, 1992. 23778593 10.1038/ncomms2992

[mol212615-bib-0003] Black JD , Lopez S , Cocco E , Schwab CL , English DP and Santin AD (2015) *Clostridium perfringens* enterotoxin (CPE) and CPE‐binding domain (c‐CPE) for the detection and treatment of gynecologic cancers. Toxins (Basel) 7, 1116–1125. 25835384 10.3390/toxins7041116 PMC4417958

[mol212615-bib-0004] Bocsik A , Walter FR , Gyebrovszki A , Fulop L , Blasig I , Dabrowski S , Otvos F , Toth A , Rakhely G , Veszelka S *et al* (2016) Reversible opening of intercellular junctions of intestinal epithelial and brain endothelial cells with tight junction modulator peptides. J Pharm Sci 105, 754–765. 26869428 10.1016/j.xphs.2015.11.018

[mol212615-bib-0005] Boireau S , Buchert M , Samuel MS , Pannequin J , Ryan JL , Choquet A , Chapuis H , Rebillard X , Avances C , Ernst M *et al* (2007) DNA‐methylation‐dependent alterations of claudin‐4 expression in human bladder carcinoma. Carcinogenesis 28, 246–258. 16829686 10.1093/carcin/bgl120

[mol212615-bib-0006] Briggs DC , Naylor CE , Smedley JG III , Lukoyanova N , Robertson S , Moss DS , McClane BA and Basak AK (2011) Structure of the food‐poisoning *Clostridium perfringens* enterotoxin reveals similarity to the aerolysin‐like pore‐forming toxins. J Mol Biol 413, 138–149. 21839091 10.1016/j.jmb.2011.07.066 PMC3235586

[mol212615-bib-0007] Casagrande F , Cocco E , Bellone S , Richter CE , Bellone M , Todeschini P , Siegel E , Varughese J , Arin‐Silasi D , Azodi M *et al* (2011) Eradication of chemotherapy‐resistant CD44+ human ovarian cancer stem cells in mice by intraperitoneal administration of *Clostridium perfringens* enterotoxin. Cancer 117, 5519–5528. 21692061 10.1002/cncr.26215 PMC3701957

[mol212615-bib-0008] Chakrabarti G and McClane BA (2005) The importance of calcium influx, calpain and calmodulin for the activation of CaCo‐2 cell death pathways by *Clostridium perfringens* enterotoxin. Cell Microbiol 7, 129–146. 15617529 10.1111/j.1462-5822.2004.00442.x

[mol212615-bib-0009] Corsini M , Ravaggi A , Odicino F , Santin AD , Ravelli C , Presta M , Romani C and Mitola S (2018) Claudin3 is localized outside the tight junctions in human carcinomas. Oncotarget 9, 18446–18453. 29719617 10.18632/oncotarget.24858 PMC5915084

[mol212615-bib-0010] Dang LH , Bettegowda C , Huso DL , Kinzler KW and Vogelstein B (2001) Combination bacteriolytic therapy for the treatment of experimental tumors. Proc Natl Acad Sci USA 98, 15155–15160. 11724950 10.1073/pnas.251543698 PMC64999

[mol212615-bib-0011] Ebihara C , Kondoh M , Hasuike N , Harada M , Mizuguchi H , Horiguchi Y , Fujii M and Watanabe Y (2006) Preparation of a claudin‐targeting molecule using a C‐terminal fragment of *Clostridium perfringens* enterotoxin. J Pharmacol Exp Ther 316, 255–260. 16183701 10.1124/jpet.105.093351

[mol212615-bib-0012] Eichner M , Augustin C , Fromm A , Piontek A , Walther W , Bucker R , Fromm M , Krause G , Schulzke JD , Gunzel D *et al* (2017) In colon epithelia, *Clostridium perfringens* enterotoxin causes focal leaks by targeting claudins which are apically accessible due to tight junction derangement. J Infect Dis 217, 147–157. 28968861 10.1093/infdis/jix485

[mol212615-bib-0013] Fischer AH , Jacobson KA , Rose J and Zeller R (2008) Hematoxylin and eosin staining of tissue and cell sections. CSH Protoc 3. doi: 10.1101/pdb.prot073411 21356829

[mol212615-bib-0014] Fujita K , Katahira J , Horiguchi Y , Sonoda N , Furuse M and Tsukita S (2000) *Clostridium perfringens* enterotoxin binds to the second extracellular loop of claudin‐3, a tight junction integral membrane protein. FEBS Lett 476, 258–261. 10913624 10.1016/s0014-5793(00)01744-0

[mol212615-bib-0015] Hashimoto Y , Shirakura K , Okada Y , Takeda H , Endo K , Tamura M , Watari A , Sadamura Y , Sawasaki T , Doi T *et al* (2017) Claudin‐5‐binders enhance permeation of solutes across the blood‐brain barrier in a mammalian model. J Pharmacol Exp Ther 363, 275–283. 28819070 10.1124/jpet.117.243014

[mol212615-bib-0016] Jian Y , Chen C , Li B and Tian X (2015) Delocalized claudin‐1 promotes metastasis of human osteosarcoma cells. Biochem Biophys Res Comm 466, 356–361. 26361141 10.1016/j.bbrc.2015.09.028

[mol212615-bib-0017] Jung JH , Jung CK , Choi HJ , Jun KH , Yoo J , Kang SJ and Lee KY (2009) Diagnostic utility of expression of claudins in non‐small cell lung cancer: different expression profiles in squamous cell carcinomas and adenocarcinomas. Pathol Res Pract 205, 409–416. 19231096 10.1016/j.prp.2008.12.015

[mol212615-bib-0018] Kitadokoro K , Nishimura K , Kamitani S , Fukui‐Miyazaki A , Toshima H , Abe H , Kamata Y , Sugita‐Konishi Y , Yamamoto S , Karatani H *et al* (2011) Crystal structure of *Clostridium perfringens* enterotoxin displays features of {beta}‐pore‐forming toxins. J Biol Chem 286, 19549–19555. 21489981 10.1074/jbc.M111.228478 PMC3103334

[mol212615-bib-0019] Kokai‐Kun JF and McClane BA (1997) Deletion analysis of the *Clostridium perfringens* enterotoxin. Infect Immun 65, 1014–1022. 9038311 10.1128/iai.65.3.1014-1022.1997 PMC175083

[mol212615-bib-0020] Kominsky SL , Tyler B , Sosnowski J , Brady K , Doucet M , Nell D , Smedley JG III , McClane B , Brem H and Sukumar S (2007) *Clostridium perfringens* enterotoxin as a novel‐targeted therapeutic for brain metastasis. Cancer Res 67, 7977–7982. 17804705 10.1158/0008-5472.CAN-07-1314

[mol212615-bib-0021] Kominsky SL , Vali M , Korz D , Gabig TG , Weitzman SA , Argani P and Sukumar S (2004) *Clostridium perfringens* enterotoxin elicits rapid and specific cytolysis of breast carcinoma cells mediated through tight junction proteins claudin 3 and 4. Am J Pathol 164, 1627–1633. 15111309 10.1016/S0002-9440(10)63721-2 PMC1615652

[mol212615-bib-0022] Kwon MJ (2013) Emerging roles of claudins in human cancer. Int J Mol Sci 14, 18148–18180. 24009024 10.3390/ijms140918148 PMC3794774

[mol212615-bib-0023] Liang ZY , Kang X , Chen H , Wang M and Guan WX (2017) Effect of *Clostridium perfringens* enterotoxin on gastric cancer cells SGC7901 which highly expressed claudin‐4 protein. World J Gastrointest Oncol 9, 153–159. 28451062 10.4251/wjgo.v9.i4.153 PMC5390300

[mol212615-bib-0024] Long H , Crean CD , Lee WH , Cummings OW and Gabig TG (2001) Expression of *Clostridium perfringens* enterotoxin receptors claudin‐3 and claudin‐4 in prostate cancer epithelium. Can Res 61, 7878–7881. 11691807

[mol212615-bib-0025] Lu Z , Ding L , Lu Q and Chen YH (2013) Claudins in intestines: distribution and functional significance in health and diseases. Tissue Barriers 1, e24978. 24478939 10.4161/tisb.24978 PMC3879173

[mol212615-bib-0026] McClane BA (2001) The complex interactions between *Clostridium perfringens* enterotoxin and epithelial tight junctions. Toxicon 39, 1781–1791. 11595640 10.1016/s0041-0101(01)00164-7

[mol212615-bib-0027] Michl P , Buchholz M , Rolke M , Kunsch S , Lohr M , McClane B , Tsukita S , Leder G , Adler G and Gress TM (2001) Claudin‐4: a new target for pancreatic cancer treatment using *Clostridium perfringens* enterotoxin. Gastroenterology 121, 678–684. 11522752 10.1053/gast.2001.27124

[mol212615-bib-0028] Michl P and Gress TM (2004) Bacteria and bacterial toxins as therapeutic agents for solid tumors. Curr Cancer Drug Targets 4, 689–702. 15578923 10.2174/1568009043332727

[mol212615-bib-0029] Minton NP (2003) Clostridia in cancer therapy. Nat Rev Microbiol 1, 237–242. 15035028 10.1038/nrmicro777

[mol212615-bib-0030] Morin PJ (2005) Claudin proteins in human cancer: promising new targets for diagnosis and therapy. Can Res 65, 9603–9606. 10.1158/0008-5472.CAN-05-2782 16266975

[mol212615-bib-0031] Morin PJ (2007) Claudin proteins in ovarian cancer. Dis Markers 23, 453–457. 18057528 10.1155/2007/674058 PMC3851116

[mol212615-bib-0032] Nemeth J , Nemeth Z , Tatrai P , Peter I , Somoracz A , Szasz AM , Kiss A and Schaff Z (2010) High expression of claudin‐1 protein in papillary thyroid tumor and its regional lymph node metastasis. Pathol Oncol Res 16, 19–27. 19578981 10.1007/s12253-009-9182-9

[mol212615-bib-0033] Neuhaus W , Piontek A , Protze J , Eichner M , Mahringer A , Subileau EA , Lee IM , Schulzke JD , Krause G and Piontek J (2018) Reversible opening of the blood‐brain barrier by claudin‐5‐binding variants of *Clostridium perfringens* enterotoxin's claudin‐binding domain. Biomaterials 161, 129–143. 29421550 10.1016/j.biomaterials.2018.01.028

[mol212615-bib-0034] Pahle J , Menzel L , Niesler N , Kobelt D , Aumann J , Rivera M and Walther W (2017) Rapid eradication of colon carcinoma by *Clostridium perfringens* enterotoxin suicidal gene therapy. BMC Cancer 17, 129. 28193196 10.1186/s12885-017-3123-x PMC5307849

[mol212615-bib-0035] Paschoud S , Bongiovanni M , Pache J‐C and Citi S (2007) Claudin‐1 and claudin‐5 expression patterns differentiate lung squamous cell carcinomas from adenocarcinomas. Mod Pathol 20, 947–954. 17585317 10.1038/modpathol.3800835

[mol212615-bib-0036] Protze J , Eichner M , Piontek A , Dinter S , Rossa J , Blecharz KG , Vajkoczy P , Piontek J and Krause G (2015) Directed structural modification of *Clostridium perfringens* enterotoxin to enhance binding to claudin‐5. Cell Mol Life Sci 72, 1417–1432. 25342221 10.1007/s00018-014-1761-6 PMC11113963

[mol212615-bib-0037] Ramalingam S and Belani C (2008) Systemic chemotherapy for advanced non‐small cell lung cancer: recent advances and future directions. Oncologist 13(Suppl 1), 5–13. 18263769 10.1634/theoncologist.13-S1-5

[mol212615-bib-0038] Robertson SL , Smedley JG III and McClane BA (2010) Identification of a claudin‐4 residue important for mediating the host cell binding and action of *Clostridium perfringens* enterotoxin. Infect Immun 78, 505–517. 19884339 10.1128/IAI.00778-09 PMC2798200

[mol212615-bib-0039] Santin AD , Bellone S , Siegel ER , McKenney JK , Thomas M , Roman JJ , Burnett A , Tognon G , Bandiera E and Pecorelli S (2007) Overexpression of *Clostridium perfringens* enterotoxin receptors claudin‐3 and claudin‐4 in uterine carcinosarcomas. Clin Cancer Res 13, 3339–3346. 17545541 10.1158/1078-0432.CCR-06-3037

[mol212615-bib-0040] Santin AD , Cane S , Bellone S , Palmieri M , Siegel ER , Thomas M , Roman JJ , Burnett A , Cannon MJ and Pecorelli S (2005) Treatment of chemotherapy‐resistant human ovarian cancer xenografts in C.B‐17/SCID mice by intraperitoneal administration of clostridium perfringens enterotoxin. Can Res 65, 4334–4342. 10.1158/0008-5472.CAN-04-3472 15899825

[mol212615-bib-0041] Shinoda T , Shinya N , Ito K , Ohsawa N , Terada T , Hirata K , Kawano Y , Yamamoto M , Kimura‐Someya T , Yokoyama S *et al* (2016) Structural basis for disruption of claudin assembly in tight junctions by an enterotoxin. Sci Rep 6, 33632. 27647526 10.1038/srep33632 PMC5028891

[mol212615-bib-0042] Silva AP , Coelho PV , Anazetti M and Simioni PU (2017) Targeted therapies for the treatment of non‐small‐cell lung cancer: monoclonal antibodies and biological inhibitors. Hum Vaccin Immunother 13, 843–853. 27831000 10.1080/21645515.2016.1249551 PMC5404364

[mol212615-bib-0043] Singh P , Toom S and Huang Y (2017) Anti‐claudin 18.2 antibody as new targeted therapy for advanced gastric cancer. J Hematol Oncol 10, 105. 28494772 10.1186/s13045-017-0473-4 PMC5427576

[mol212615-bib-0044] Soini Y and Talvensaari‐Mattila A (2006) Expression of claudins 1, 4, 5, and 7 in ovarian tumors of diverse types. Int J Gynecol Pathol 25, 330–335. 16990707 10.1097/01.pgp.0000215298.38114.cc

[mol212615-bib-0045] Sonoda N , Furuse M , Sasaki H , Yonemura S , Katahira J , Horiguchi Y and Tsukita S (1999) *Clostridium perfringens* enterotoxin fragment removes specific claudins from tight junction strands: evidence for direct involvement of claudins in tight junction barrier. J Cell Biol 147, 195–204. 10508866 10.1083/jcb.147.1.195 PMC2164970

[mol212615-bib-0046] Sun BS , Yao YQ , Pei BX , Zhang ZF and Wang CL (2016) Claudin‐1 correlates with poor prognosis in lung adenocarcinoma. Thorac Cancer 7, 556–563. 27766775 10.1111/1759-7714.12368 PMC5130200

[mol212615-bib-0047] Takahashi A , Saito Y , Kondoh M , Matsushita K , Krug SM , Suzuki H , Tsujino H , Li X , Aoyama H , Matsuhisa K *et al* (2012) Creation and biochemical analysis of a broad‐specific claudin binder. Biomaterials 33, 3464–3474. 22317861 10.1016/j.biomaterials.2012.01.017

[mol212615-bib-0048] Tanaka S , Aoyama T , Ogawa M , Takasawa A , Murata M , Osanai M , Saito T and Sawada N (2018) Cytotoxicity of *Clostridium perfringens* enterotoxin depends on the conditions of claudin‐4 in ovarian carcinoma cells. Exp Cell Res 371, 278–286. 30142326 10.1016/j.yexcr.2018.08.024

[mol212615-bib-0049] Veshnyakova A , Krug SM , Mueller SL , Piontek J , Protze J , Fromm M and Krause G (2012a) Determinants contributing to claudin ion channel formation. Ann N Y Acad Sci 1257, 45–53. 22671588 10.1111/j.1749-6632.2012.06566.x

[mol212615-bib-0050] Veshnyakova A , Piontek J , Protze J , Waziri N , Heise I and Krause G (2012b) Mechanism of *Clostridium perfringens* enterotoxin interaction with claudin‐3/‐4 protein suggests structural modifications of the toxin to target specific claudins. J Biol Chem 287, 1698–1708. 22128179 10.1074/jbc.M111.312165 PMC3265853

[mol212615-bib-0051] Veshnyakova A , Protze J , Rossa J , Blasig I , Krause G and Piontek J (2010) On the interaction of *Clostridium perfringens* enterotoxin with claudins. Toxins 2, 1336–1356. 22069641 10.3390/toxins2061336 PMC3153257

[mol212615-bib-0052] Walther W , Petkov S , Kuvardina ON , Aumann J , Kobelt D , Fichtner I , Lemm M , Piontek J , Blasig IE , Stein U *et al* (2012) Novel *Clostridium perfringens* enterotoxin suicide gene therapy for selective treatment of claudin‐3‐ and ‐4‐overexpressing tumors. Gene Ther 19, 494–503. 21975465 10.1038/gt.2011.136

[mol212615-bib-0053] Wartofsky L (2010) Increasing world incidence of thyroid cancer: increased detection or higher radiation exposure? Hormones (Athens) 9, 103–108. 20687393 10.14310/horm.2002.1260

[mol212615-bib-0054] Winkler L , Gehring C , Wenzel A , Muller SL , Piehl C , Krause G , Blasig IE and Piontek J (2009) Molecular determinants of the interaction between *Clostridium perfringens* enterotoxin fragments and claudin‐3. J Biol Chem 284, 18863–18872. 19429681 10.1074/jbc.M109.008623 PMC2707212

[mol212615-bib-0055] Yoda S , Soejima K , Hamamoto J , Yasuda H , Nakayama S , Satomi R , Terai H , Ikemura S , Sato T , Naoki K *et al* (2014) Claudin‐1 is a novel target of miR‐375 in non‐small‐cell lung cancer. Lung Cancer 85, 366–372. 25001509 10.1016/j.lungcan.2014.06.009

[mol212615-bib-0056] Zwanziger D , Badziong J , Ting S , Moeller LC , Schmid KW , Siebolts U , Wickenhauser C , Dralle H and Fuehrer D (2015) The impact of CLAUDIN‐1 on follicular thyroid carcinoma aggressiveness. Endocr Relat Cancer 22, 819–830. 26219679 10.1530/ERC-14-0502

