# Supplementary material for: Targeting claudin‐overexpressing thyroid and lung cancer by modified Clostridium perfringens enterotoxin
Source: Mol Oncol. 2020 Jan 8;14(2):261–76. doi: 10.1002/1878-0261.12615 (PMC6998413; doi:10.1002/1878-0261.12615)
Supplement: Supplementary file 1 — Fig. S1. Shift of claudin‐subtype‐directed cytotoxicity of CPE by structure‐guided mutation of CPE. Fig. S2. Claudin‐subtype‐directed cytotoxicity of CPE variants is mediated by induction of Ca2+ influx. Fig. S3. Visualization of CPE and claudins in thyroid and lung xenograft models after recombinant CPE treatment. Table S1. cCPE or CPE variants and claudin‐binding characteristics. [file MOL2-14-261-s001.pdf]

Supplemental Material

Targeting claudin-overexpressing thyroid and lung cancer by modified *Clostridium perfringens* enterotoxin

Molecular Oncology

Anna Piontek, Miriam Eichner, Denise Zwanziger, Laura-Sophie Beier, Jonas Protze, Wolfgang Walther, Sarah Theurer, Kurt Werner Schmid, Dagmar Führer-Sakel, Jörg Piontek[#] , Gerd Krause[#]

# Corresponding authors: Gerd Krause, Robert-Rössle-Str. 10, 13125 Berlin, Germany; Tel +4930 94793 228, GKrause@fmp-berlin.de; Jörg Piontek, Hindenburgdamm 30, 12167 Berlin, Germany; Tel +4930 450-514535; joerg.piontek@charite.de

Table S1. **cCPE- or CPE-variants and claudin-binding characteristics**

**Mutations in cCPE or CPE**	**Abbreviation in this study**	**Claudin sensitivity***	**Published as cCPE-variant**
No mutations	wt	Cldn3,-4,-7,-8,-9	(1-4)
Y306A/L315A	Negative control	Not sensitive	(4)
S313H	Mut1	Cldn1,-3,-4,-5,-6,-7,-9	(4)
S231R	Mut2		
S231R/S313H	Mut3	Cldn1,-5	
S305P/S307R/S313H	Mut4	Cldn1-6	(4)
S231R/Y306W/S313H	Mut5		
N218Q/Y306W/S313H	Mut6	Cldn5	(2)
N218Q/S231R/S313H	Mut7		
N218Q/S231R/Y306W/S313H	Mut8		
Y306W/S313H	Mut9	Cldn3,-4, -5	(2,4)
L254A/S256A/I258A/D284A	Mut10	Cldn4	(3)

Mutations introduced in full length CPE or its C-terminal domain (cCPE) result in different CPE/cCPE-variants with modified claudin-sensitivity. In the table the strongest binding partners (claudin subtypes, *) of these variants are shown.

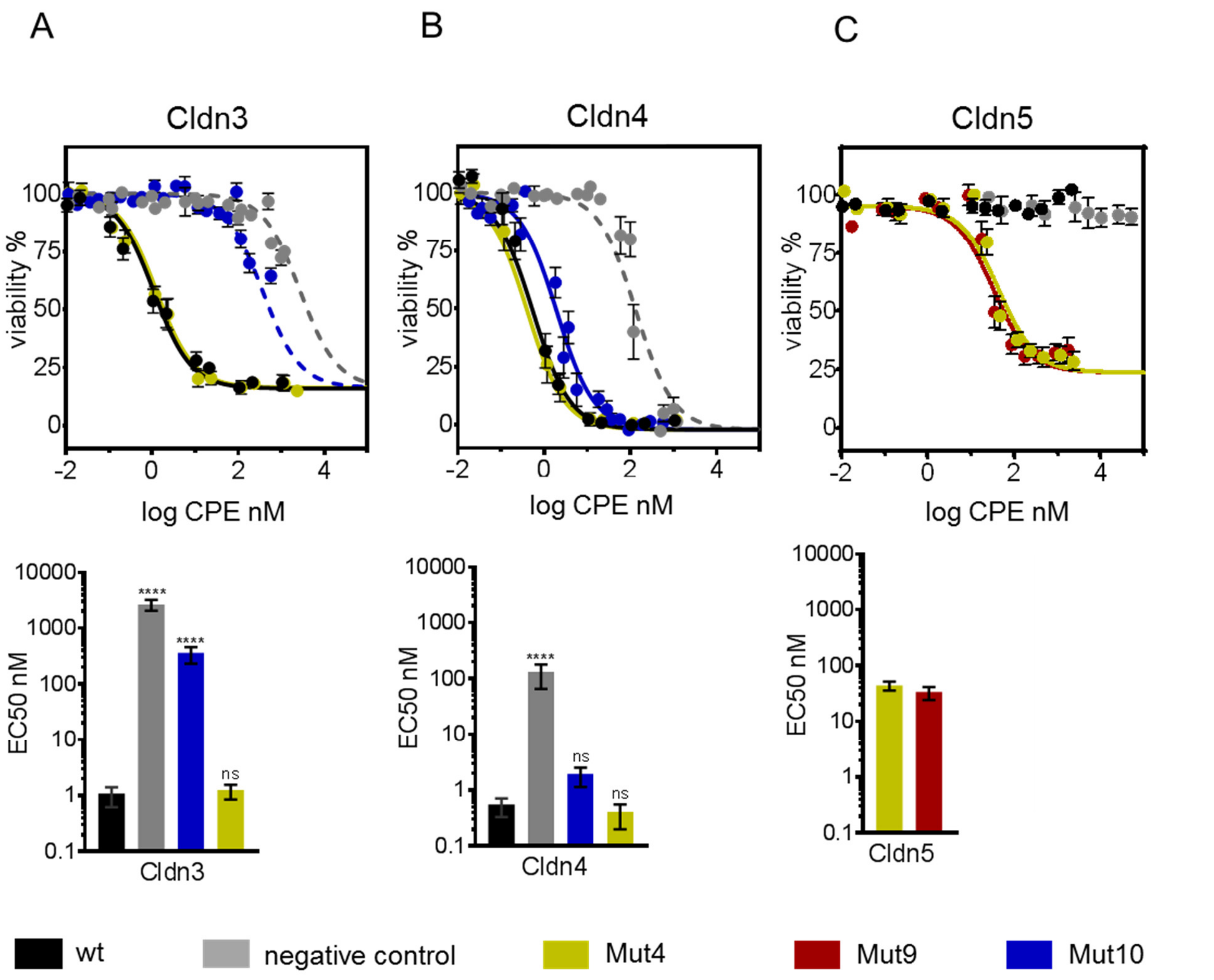

Fig. S1 **Shift of claudin subtype-directed cytotoxicity of CPE by structure-guided mutation of CPE**. (**A, B**) L254A/S256A/I258A/S284A (Mut10) substitutions decreases cytotoxicity of CPE to Cldn3-expressing HEK cells but not to Cldn4-expressing HEK cells. (**C**) Y306W/S313H (Mut9) and S305P/S307R/S313H (Mut4) substitutions enable Cldn5-directed cytotoxicity of CPE. (**A**) Cldn3-YFP (Cldn3), (**B**) Cldn4-FLAG- (Cldn4) and (**C**) Cldn5-YFP- (Cldn5)- stably expressing HEK cells were incubated with different concentrations of CPE-variants for 1h and viability analysed by MTT-assays. logEC50 values were calculated with GraphPad Prism 6 using the model "log(agonist) vs. response" and dose-response curve was plotted using the EC50 value as described previously (1). For EC-50 values, mean $\pm$ SEM, N ≥ 5, One-way ANOVA with Bonferroni correction. ns, not significant vs wt ($p > 0.05$); **** , p≤0,0001 vs wt.

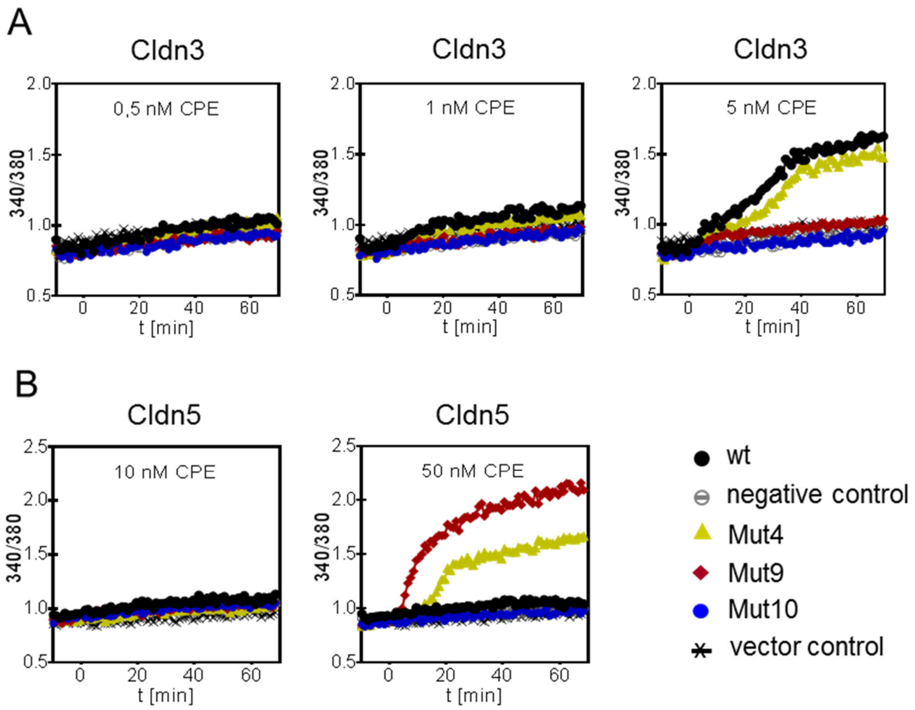

Fig. S2 **Claudin-subtype-directed cytotoxicity of CPE-variants is mediated by induction of Ca^{2+} influx. (A)** CPEwt and CPE-Mut4 but not CPE-Mut10, CPE-Mut9, CPE-negative control or vector control mediate concentration-dependent Ca^{2+}-influx in Cldn3-YFP-expressing HEK cells. (**B**) CPE-Mut9 and CPE-Mut4 but not CPEwt, CPE-Mut10 or vector control mediate concentration-dependent Ca^{2+}-influx in Cldn5-YFP-expressing HEK cells. Stable lines were at time point 0 incubated with different concentrations of CPE-variants and Ca^{2+}-influx was measured over time with Fura-2 as described earlier (1).The ratio of emission at 510 nm after 340 nm and 380 nm excitation (340/380) reflects the intracellular Ca^{2+}-level.

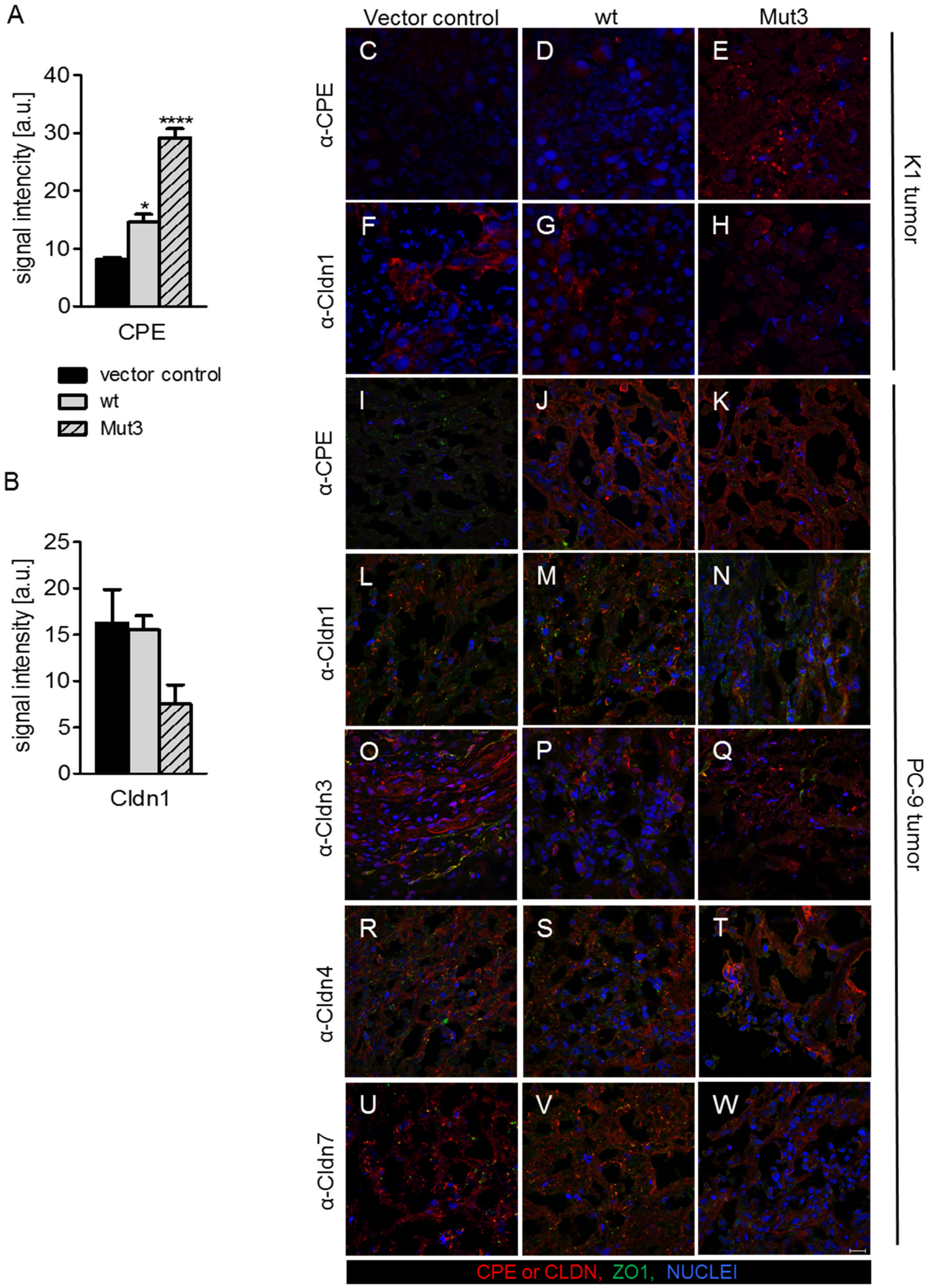
A
signal intencity [a.u.]
40
30
20
10
0
*
****
CPE
vector control
wt
Mut3
B
signal intensity [a.u.]
25
20
15
10
5
0
Cldn1
Vector control
wt
Mut3
C
D
E
F
G
H
I
J
K
L
M
N
O
P
Q
R
S
T
U
V
W
α-CPE
α-Cldn1
α-CPE
α-Cldn1
α-Cldn3
α-Cldn4
α-Cldn7
K1 tumor
PC-9 tumor
CPE or CLDN, ZO1, NUCLEI

Fig. S3 **Visualization of CPE and claudins in thyroid and lung xenograft models after recombinant CPE treatment**. In K1 xenograft model strongest CPE-signal was detected in CPE-Mut3 treated samples (**A**), on the contrary these samples revealed weakest Cldn1- signal (**B**). This might point to the stronger internalization and degradation of Cldn1 after binding of CPE-Mut3. Analysis revealed no junctional claudin or ZO-1 staining in K1 (**F-H**) or PC-9 tumors (**I-W**), pointing to absence of intact TJ in the thyroid and lung tumor xenograft models. CPEwt and -Mut3 treatment led to a slight decrease of Cldn3,-4 and -7- signal through potential internalization and degradation of those claudins after interaction with CPE. 18 µm sick slices of shock frozen tumor samples were stained for analysis of potential claudin-disarrangement after CPE-treatment. Lung tumor samples were stained with mouse anti-ZO1 and goat anti-mouse Alexa 488 antibodies for visualization of ZO1 as junctional marker (green). DAPI staining for nuclei (blue) was performed for all samples, as well. CPE (**C-E**, **I-K**), Cldn1 (**F-H**, **L-N**), Cldn3 (**O-Q**), Cldn4 (**R-T**) or Cldn7 (**U-W**) were visualized (red) by incubation with rabbit anti-CPE or rabbit anti-claudin (either Cldn1, -3, -4 or -7) antibody, followed by incubation with goat anti-rabbit Alexa 555 antibodies. Scale bar of 20 µm.

References

1. Eichner, M., Augustin, C., Fromm, A., Piontek, A., Walther, W., Bucker, R., Fromm, M., Krause, G., Schulzke, J. D., Gunzel, D., and Piontek, J. (2017) In Colon Epithelia, Clostridium perfringens Enterotoxin Causes Focal Leaks by Targeting Claudins Which are Apically Accessible Due to Tight Junction Derangement. *The Journal of infectious diseases* **217**, 147-157
2. Neuhaus, W., Piontek, A., Protze, J., Eichner, M., Mahringer, A., Subileau, E. A., Lee, I. M., Schulzke, J. D., Krause, G., and Piontek, J. (2018) Reversible opening of the blood-brain barrier by claudin-5-binding variants of Clostridium perfringens enterotoxin's claudin-binding domain. *Biomaterials* **161**, 129-143
3. Veshnyakova, A., Piontek, J., Protze, J., Waziri, N., Heise, I., and Krause, G. (2012) Mechanism of Clostridium perfringens enterotoxin interaction with claudin-3/-4 protein suggests structural modifications of the toxin to target specific claudins. *J Biol Chem* **287**, 1698-1708
4. Protze, J., Eichner, M., Piontek, A., Dinter, S., Rossa, J., Blecharz, K. G., Vajkoczy, P., Piontek, J., and Krause, G. (2015) Directed structural modification of Clostridium perfringens enterotoxin to enhance binding to claudin-5. *Cell Mol Life Sci* **72**, 1417-1432
